# Agreement between heuristic shrinkage factor and optimal shrinkage factors in logistic regression for risk prediction: a simulation study across different sample sizes and settings

**DOI:** 10.1186/s41512-026-00222-1

**Published:** 2026-05-18

**Authors:** Alexander Pate, Glen P. Martin, Richard D. Riley

**Affiliations:** 1 Centre for Informatics, Imaging and Data Science, Faculty of Biology, Medicine and Health, University of Manchester, Manchester, UK; 2 Department of Applied Health Sciences, School of Health Sciences, College of Medicine and Health, University of Birmingham, Birmingham, B15 2TT UK; 3 National Institute for Health and Care Research (NIHR) Birmingham Biomedical Research Centre, Birmingham, UK

## Abstract

**Introduction:**

The heuristic shrinkage factor of Van Houwelingen and Le Cessie (𝑆_𝑉𝐻_) is a commonly used closed-form solution to adjust for overfitting in unpenalised logistic regression models for risk prediction. It is also the basis of widely-adopted minimum sample size criteria for developing clinical prediction models. However, current evidence is lacking regarding the bias of 𝑆_𝑉𝐻_ compared to the optimal shrinkage factor (𝑆_𝑜𝑝𝑡_). Here, we examine this issue and also assess the bias of an alternative bootstrap-derived shrinkage factor (𝑆_𝑏𝑜𝑜𝑡_).

**Methods:**

We undertook two simulation studies. The first examined the bias of 𝑆_𝑉𝐻_ and 𝑆_𝑏𝑜𝑜𝑡_ as estimators of 𝑆_𝑜𝑝𝑡_ across a range of different scenarios defined by 𝐶_𝑝𝑜𝑝_, the C-statistic of the model developed in a population sized dataset. The second examined the bias of 𝑆_𝑜𝑝𝑡_ when using development sample sizes targeting a shrinkage of 0.9, based on a sample size calculation defined by 𝑆_𝑉𝐻_ itself (𝑁_𝑜𝑟𝑖𝑔𝑖𝑛𝑎𝑙_) or by an adapted simulation-based approach (𝑁_𝑠𝑖𝑚_).

**Results:**

For high C-statistics, 𝑆_𝑉𝐻_ overestimated 𝑆_𝑜𝑝𝑡_, whereas for low C-statistics 𝑆_𝑉𝐻_ underestimates 𝑆_𝑜𝑝𝑡_. For example, across scenarios when 0.8≤𝐶_𝑝𝑜𝑝_<0.85, the 95-percentile range in the bias was (0.005,0.387), compared to (−0.580,−0.007) across scenarios when 0.6≤𝐶_𝑝𝑜𝑝_<0.65. The magnitude of bias increased as 𝐶_𝑝𝑜𝑝_ tended to either 0.5 or 1. As sample size increased and 𝑆_𝑜𝑝𝑡_→1, the magnitude of the bias in either direction reduced. 𝑆_𝑏𝑜𝑜𝑡_ was less biased than 𝑆_𝑉𝐻_, with a median magnitude of bias across all scenarios of 0.007, compared to 0.032 for 𝑆_𝑉𝐻_. Developing models on datasets of size 𝑁_𝑠𝑖𝑚_ gave 𝑚𝑒𝑎𝑛(𝑆_𝑜𝑝𝑡_) closer to 0.9 (mean magnitude of bias across all scenarios 0.004) than 𝑁_𝑜𝑟𝑖𝑔𝑖𝑛𝑎𝑙_ (mean magnitude of bias 0.041).

**Conclusions:**

𝑆_𝑉𝐻_ is often a poor estimator of the optimal global shrinkage factor. If global shrinkage is needed, we recommend using the bootstrap shrinkage estimate. The bootstrap estimate shows minimal bias in most scenarios, though in small samples the variability is large so provides no guarantees to address overfitting in a single dataset. A sample size calculation based on simulation is often preferable over formula dependent on targeting 𝑆_𝑉𝐻_.

**Supplementary Information:**

The online version contains supplementary material available at 10.1186/s41512-026-00222-1.

## Introduction

Clinical prediction models are used to estimate the risk of a future event (prognostic), or the presence of an existing condition (diagnostic) [1]. When developing a prediction model, a key concern is overfitting to the development dataset, which leads to predictions that are too extreme (e.g., estimated risks are too low for some individuals, and too high for other individuals) in new data from the same population. In a regression framework, penalisation and shrinkage techniques are available to help mitigate against this issue. One approach, that is often used in practice, is to fit a standard (unpenalised) regression model and then apply a post-estimation global shrinkage factor ($S$) to all the predictor effect estimates, to ‘shrink’ predicted values closer to the mean. The optimal value of $S$ is such that after it has been applied, the calibration slope of the adjusted model will be 1 in the target population of interest [2] (this is the value of S which minimises the expected mean-square error of predictions from the model in the target population of interest [3]). We refer to this optimal value as ${S}_{opt}$; when developing a model ${S}_{opt}$ is unknown and must be estimated.

Previous research has established that estimators of ${S}_{opt}$ have some issues. Van Houwelingen observed that a variety of shrinkage estimators were negatively correlated with the optimal amount of shrinkage [4]. This finding was verified for a range of shrinkage techniques in a more comprehensive simulation study by van Calster et al. [5] Both van Calster et al., [5] and Riley et al., [3] showed that there was high variability in the calibration slope of models with shrinkage techniques applied, particularly when sample size was small. Following this, Martin et al., [6] highlighted the importance of quantifying variability in predictive performance of model with shrinkage techniques applied, even when models meet minimum sample size requirements. All of these studies highlight that, in a given dataset, shrinkage estimators will often give a poor estimate of the optimal amount of shrinkage required, i.e., there is a high amount of variability in the performance of shrinkage estimators. On the contrary, they also show that the shrinkage estimators improve calibration in new data compared to unpenalised approaches *on average*, with mean calibration slopes closer to 1 in new data. Despite this improvement, these simulation results indicate that the shrinkage estimates provide a $mean({S}_{opt})$ that is not close to 1 in some scenarios, and that further research is required to examine and potentially improve estimators of ${S}_{opt}$* on average. *A good estimator should perform well on average, i.e., either unbiased, or small levels of bias, to ensure it does not consistently over or underestimate the quantity of interest.

For binary logistic regression models, van Houwelingen and Le Cessie [7] proposed a closed form estimate of the global shrinkage factor; the “heuristic” shrinkage factor, ${S}_{VH}$. With increases in computing power, bootstrapping, ${S}_{boot}$, has also become a viable method for estimating $S$after fitting a standard (unpenalised) regression model [2, 8, 9]. The mathematical details are presented in Key definitions. The heuristic shrinkage factor is commonly used to assess the level of or adjust for optimism post model fitting [10–16], and has been embedded within widely-used sample size criteria for model development (to be used before data collection or modelling begins) for binary logistic and Cox regression [17]. In short, one identifies the minimum sample size that targets a particular ${S}_{VH}$ (such as 0.9 or above, reflecting low overfitting), based on assumed values of other parameters in the closed-form solution; this is not possible for ${S}_{boot}$, which is only applicable post-model fitting. Sample size criteria are an important determinant of the quality of AI-based prediction models [18].

Given the considerable use of ${S}_{VH}$ in practice (both before and during analysis), and previous methodology evidence that raises doubts as to its performance, it is important to firmly establish the reliability of ${S}_{VH}$ as an estimator for ${S}_{opt}$ on average, which one would expect as a minimum requirement of an estimator. Therefore, in this article we have two aims:

1. To extensively compare the performance of ${S}_{VH}$ and ${S}_{boot}$ as estimators for ${S}_{opt}$ on average, and attempt to identify in what settings ${S}_{VH}$ is a biased estimator of ${S}_{opt}$.
2. To examine the impact of any bias in ${S}_{VH}$ on the sample size criteria of Riley et al., [17] and compare performance with a recently proposed simulation-based adjustment of Pavlou et al. [19]

These aims are achieved through two simulation studies, described in “Simulation study 1: Examine performance of ${\widehat{{\boldsymbol{S}}}}_{{\boldsymbol{V}}{\boldsymbol{H}}}$ and ${\widehat{{\boldsymbol{S}}}}_{{\boldsymbol{b}}{\boldsymbol{o}}{\boldsymbol{o}}{\boldsymbol{t}}}$ as estimators of ${{\boldsymbol{S}}}_{{\boldsymbol{o}}{\boldsymbol{p}}{\boldsymbol{t}}}$” section and “Simulation Study 2: Example performance of the Riley et al. [18] sample size criteria using original or Pavlou adjustment [20]” section respectively, followed by examples to showcase the impact of the results on applied research in “Impact of using a different data generating mechanism” section. “Examples to demonstrate impact of findings” section concludes with discussion and recommendations.

### Key definitions

Consider there is a development dataset available with outcome ${Y}_{i}$ and a vector of $Q$ predictor variables ${{\boldsymbol{X}}}_{{\boldsymbol{i}}}=\left({X}_{1i},{X}_{2i},\dots ,{X}_{Qi}\right)$ for individual $i$. We want to develop a prediction model using this dataset, via a logistic regression model containing an intercept term and multiple predictor effects. If this logistic regression model is estimated without shrinkage (i.e., using traditional maximum likelihood estimation), then post-estimation, a global shrinkage factor ($S$) can be applied to all predictor effects which shrinks predicted risks towards the mean. Specifically, one multiplies the unpenalised (e.g. standard maximum likelihood estimates) ${\widehat{\beta }}_{1},\dots ,{\widehat{\beta }}_{Q}$ by $S$, giving,

1 $$\mathrm{log}\left(\frac{P\left({Y}_{i}=1|{X}_{i}\right)}{1-P\left({Y}_{i}=1|{X}_{i}\right)}\right)={\alpha }^{*}+{S(\widehat{\beta }}_{1}{X}_{1i}+\dots +{\beta }_{Q}{X}_{Qi}),$$

where ${\alpha }^{*}$ is the revised intercept to ensure the appropriate mean predicted risk in the population of interest. We now drop the subscript $i$ for brevity.

The optimal shrinkage (${S}_{opt}$) is the value of $S$ such that the adjusted model has a calibration slope of 1 when validated in the population of interest. If a large validation cohort representative of the population is available, ${S}_{opt}$ can be estimated by calculating the calibration slope in this dataset:

2 $$\mathrm{log}\left(\frac{P(Y=1)}{1-P(Y=1)}\right)={\alpha }^{*}+{S}_{opt}*logit\left(\widehat{p}\right),$$

where $\widehat{p}$ is the estimated risk derived using Eq. (1) developed on the development dataset. The validation dataset should be large enough that sampling the dataset is almost the same as sampling from the population

In the absence of such a large dataset, then ${S}_{opt}$ can be estimated by the heuristic shrinkage factor of Van Houwelingen and Le Cessie [7] as:

3 $${\widehat{S}}_{VH}=1-\frac{Q}{LR} ,$$

where $Q$ is the number of predictor parameters (excluding intercept), and $LR=-2({\mathit{lnL}}_{\mathit{null}}-\mathit{ln}{L}_{\mathit{model}})$ is the likelihood ratio statistic

It can also be estimated by the bootstrap-derived uniform shrinkage factor [8, 20] (${\widehat{S}}_{boot}$), calculated as follows:

• sampling (with replacement) a large number of bootstrap datasets (usually > 200) from the development dataset

• fitting the model of interest (using the same development process as done for the original model) in each bootstrap dataset

• calculating the calibration slope of each bootstrap model in the original development dataset

• calculating the uniform shrinkage factor, ${\widehat{S}}_{boot}$, as the average of these calibration slopes across all the bootstrap models

## Simulation study 1: Examine performance of ${\widehat{{\boldsymbol{S}}}}_{{\boldsymbol{V}}{\boldsymbol{H}}}$ and ${\widehat{{\boldsymbol{S}}}}_{{\boldsymbol{b}}{\boldsymbol{o}}{\boldsymbol{o}}{\boldsymbol{t}}}$ as estimators of ${{\boldsymbol{S}}}_{{\boldsymbol{o}}{\boldsymbol{p}}{\boldsymbol{t}}}$

The simulation follows the aims, data generating mechanisms, estimands, methods, performance metrics (ADEMP) [21] structure.

### Aims

The aim of this simulation is to examine, across a range of different scenarios, whether ${S}_{VH}$ is a biased estimator of ${S}_{opt}$ when developing a logistic regression model.

### Data generating mechanisms

#### Data generating setup for each scenario

Multiple datasets of size ${n}_{dev}$ individuals were generated for each scenario (see below), with outcome status (${Y}_{i}=1$ event, $0$ no event) randomly sampled according to a true binary logistic regression model containing Q predictors. We considered $Q$ continuous predictor ${\boldsymbol{X}}\sim MVN(0,\Omega )$, with true regression coefficients ${\beta }_{j}$ (log odds ratios). Thus, true risks for each individual corresponded to,

$${p}_{i}=\frac{exp\left({\beta }_{0}+{X}_{1,i}{\beta }_{1}+\dots +{X}_{Q,i}{\beta }_{Q}\right)}{1+exp\left({\beta }_{0}+{X}_{1,i}{\beta }_{1}+\dots +{X}_{Q,i}{\beta }_{Q}\right)}.$$

and so, in the development dataset, each individual’s outcome was randomly sampled ${Y}_{i}\sim Bernoulli({p}_{i})$.

#### Creation of scenarios

We consider a range of scenarios, which varied according to the number of predictors, development sample size, predictor effect sizes, outcome event proportion, and so other features, as now described.

For each scenario the input parameters $Q$, ${\beta }_{j}$, $\Omega$ and ${n}_{dev}$ were generated at random (and kept fixed for each iteration of that scenario), to ensure we had a range of scenarios that were not informed by us deterministically, and to ensure areas of the parameter space were not missed systematically. First $Q$ was generated according to $Q={Q}_{meas}+{Q}_{unmeas}$, where both ${Q}_{meas}$ and ${Q}_{unmeas}$ were chosen uniformly as random integers between $1$ and $30$, and represent the number of measured and unmeasured predictors respectively. Variables were generated according to a multivariate normal distribution, with the covariance matrix generated at random as a positive semi definite matrix using the process detailed in the supplementary material. This induces random levels of covariance between all the variables. We also simulated scenarios with no covariance between the predictors using the identity matrix as the covariance matrix.

Regression coefficients were generated according to ${\beta }_{j}\sim Uniform\left(-\mathrm{0.5,0.5}\right) \forall j$*.* A mean outcome proportion was sampled according to $prop\sim Uniform\left(0.05, 0.95\right)$, and the intercept ${\beta }_{0}$ was calculated using ${\beta }_{0}=-log\left(\left(1-prop\right)/prop\right)$ in order to induce different mean outcome proportion, lying roughly between $0.05$ amd $0.95$.

The development sample size was simulated at random from distribution $\sim 100+Weibull(0.5, 2500)$. If the simulated sample size was over 5000, another draw was taken, until a sample size $\le 5000$ was achieved. This was for computational reasons, and because large sample sizes resulting in less overfitting where both ${S}_{VH}$ and ${S}_{boot}$ performed well. This resulted in development sample sizes between 100 and 5000, with smaller sample sizes simulated more frequently.

Overall, we created 15,000 scenarios using this process for data with non-zero covariance structure, and 15,000 scenarios with a zero covariance structure. Each scenario represents a different combination of all the simulation parameters mentioned above. For each scenario, we repeated the data-generating process across 250 iterations. Note that we did not consider any variable selection.

#### Process for simulation

For every scenario, with a fixed set of randomly generated input parameters, the simulation was performed as follows:

- Step 1: Generate a validation cohort of size 1,000,000 according to the process outlined in *creation of scenarios*.
- Step 2: Fit an unpenalised binary logistic regression model in the validation cohort using the first ${Q}_{meas}$ variables as predictors.
- Step 3: Estimate discrimination performance ${C}_{pop}$ (c-statistic, where 1 is perfect discrimination and 0.5 is no discrimination beyond chance), and the Cox-Snell [22] generalised definition of the apparent ${R}^{2}$ which can be epressed as: [23, 24]

$${R}_{CS\_pop}^{2}=1-exp\left(\frac{-LR}{n}\right).$$

These represent model performance that would be achieved if it were not for limited sample size.

Repeat the following steps 250 times:

- Step 4: Generate development dataset of size ${n}_{dev}$ according to the process outlined in *creation of scenarios*.
- Step 5: Develop a model by fitting an unpenalised binary logistic regression model in the development dataset, using the first ${Q}_{meas}$ variables as predictors.
- Step 6: Calculate ${\widehat{S}}_{VH}$, ${\widehat{S}}_{boot}$, ${R}_{CS\_app}^{2}$ and ${C}_{app}$ for the developed model from step 5. ${\widehat{S}}_{boot}$ is a bootstrapped estimate of the global shrinkage factor and is estimated using 200 bootstrap iterations of the development dataset. ${\widehat{S}}_{VH}$ is defined in Eq. (2) and estimated from the development dataset. ${C}_{app}$ and ${R}_{CS\_app}^{2}$ are the apparent (i.e. estimated in the development dataset) discrimination and Cox-Snell ${R}^{2}$.
- Step 7: Calculate ${S}_{opt}$ by calculating the calibration slope of the model (from step 5) in the validation cohort (from step 1).

### Estimands

The estimand is ${S}_{opt}$, the global shrinkage factor for a binary logistic regression model when it is validated in the population of interest.

### Methods

The methods of interest for estimating this estimand are ${\widehat{S}}_{VH}$ and ${\widehat{S}}_{boot}$, detailed in “Key definitions” section.

### Performance metric

Let ${\widehat{S}}_{opt}$ denote an estimator (i.e. ${\widehat{S}}_{VH}$ or ${\widehat{S}}_{boot}$). We are interested in the bias of ${\widehat{S}}_{opt}$, estimated from $mean\left({\widehat{S}}_{opt}\right)-mean({S}_{opt})$ in each simulation scenario. We viewed the bias in each scenario visually through plots, with $mean({\widehat{S}}_{opt})$ on the x-axis, and $mean({S}_{opt})$ on the y-axis. These plots were presented with respect to ${R}_{CS\_pop}^{2}, {C}_{pop}$, ${Q}_{meas}$, event per predictor parameter (EPP) and $N.$ This allows us to establish whether there is a pattern between the bias of the estimator ${\widehat{S}}_{opt}$, and properties of the model. These plots were also produced with median instead of mean.

For a numerical evaluation we report the ‘*mean bias*’, referred to as the bias moving forwards. The bias in each scenario, $k$, is estimated as:

$${bias}_{k}=mean\left({\widehat{S}}_{opt,k}\right)-mean({S}_{opt,k})=\frac{\sum\limits_{m=1}^{250}\left({\widehat{S}}_{opt,k,m}-{S}_{opt,k,m}\right)}{250},$$

where ${\widehat{S}}_{opt,k,m}$ and ${S}_{opt,k,m}$ are the estimated shrinkage factor and optimal shrinkage factor in iteration $m$ of scenario $k$. We drop the subscript $k$ for brevity when discussing these metrics, which are frequently referred to when discussing the plots.

We then report the mean, and the 2.5th, 25th, 50th, 75th and 97.5th percentiles of the bias across all the scenarios, and stratified by ${C}_{pop}$ into subgroups of scenarios. For example, when presenting the mean of the bias across a group of scenarios, this is estimated as:

$$\frac{\sum_{k\in scenariosingroup}{bias}_k}{number\;of\;scenarios\;in\;group}.$$

When referring to mean of the bias, or median of the bias, this is the mean or median of the bias across the chosen group of scenarios. We also present summary metrics for the magnitude of the bias, which is the absolute value of the bias in each scenario. For example, the mean of the magnitude of the bias is estimated as:

$$\frac{\sum_{k\in scenariosingroup}\left|{bias}_k\right|}{number\;of\;scenarios\;in\;group}.$$

We also estimated the Monte Carlo Standard Error (MCE) of the bias in each scenario $k$. First the standard deviation of the bias is calculated as:

$${sd}_{k}=\sqrt{\frac{\sum\limits_{m=1}^{250}{\left({\widehat{S}}_{opt,k,m}-{S}_{opt,k,m}-\left(\overline{\widehat{S}_{opt,k,m}-{S}_{opt,k,m}}\right)\right)}^{2}}{250}}$$

The MCE in scenario $k$ is then calculated as ${MCE}_{k}=\frac{{sd}_{k} }{\sqrt{250}}$, and we present the 50th, 90th and 99th percentiles. All plots and metrics are estimated for ${\widehat{S}}_{boot}$ as well as ${\widehat{S}}_{VH}$.

## Results

All results presented in the main manuscript consider scenarios where predictors had non-zero covariance. We simulated data for 15,000 scenarios; however some resulted in negative estimates of $mean\left({\widehat{S}}_{VH}\right), mean\left({\widehat{S}}_{boot}\right)$ and $mean\left({R}_{CS\_app}^{2}\right)$, or high values of $mean\left({\widehat{S}}_{boot}\right)>2$. These were high noise scenarios with few measured predictors, a large number of unmeasured predictors, and a ${C}_{pop}$ value close to 0.5. Other scenarios, with very high numbers of predictors and small sample size resulted in models failing to converge during the bootstrapping procedure. These were all viewed as extreme scenarios, not representative of reality, and were removed. Equivalent results for 15,000 scenarios with no covariance ($\Omega$ = identiy matrix) are presented in the supplementary material, but the same conclusions were found throughout. When presenting results, we focus on the impact of the C-statistic more so than the other metrics. This is because it is the most commonly reported metric in the literature, and was also found to be a key predictor of performance in both simulations.

**Fig. 1:**
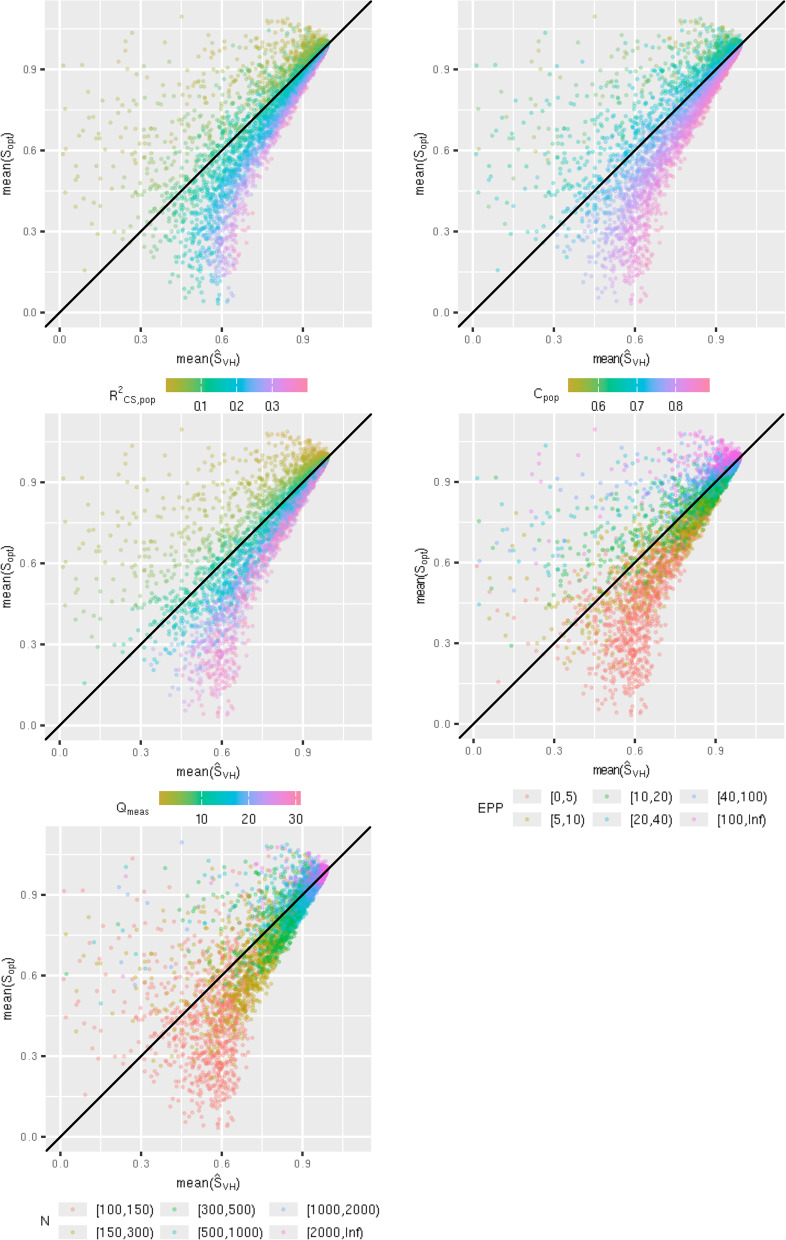
Simulation study 1: $\mathrm{mean}\left({\widehat{\mathrm{S}}}_{\mathrm{VH}}\right)$ plotted against $\mathrm{mean}({\mathrm{S}}_{\mathrm{opt}})$

Figure 1 contains $mean\left({\widehat{S}}_{VH}\right)$ plotted against $mean({S}_{opt})$ in scenarios with a non-zero covariance structure. Each data point on the plot corresponds to one scenario. In a large number of scenarios ${\widehat{S}}_{VH}$ was a poor estimator of ${S}_{opt}$. The mean magnitude of the bias was $0.077$, and the 75th percentile of the bias was 0.097 (Table 1). When ${R}_{CS\_pop}^{2}$ or ${C}_{pop}$ are higher, ${\widehat{S}}_{VH}$ overestimated ${S}_{opt}$; for example, in scenarios where $0.8\le {C}_{pop}<0.85$, the 95th percentile range of the bias was $(0.005, 0.387)$. Conversely, when they are low, ${\widehat{S}}_{VH}$ underestimates ${S}_{opt}$; for example, in scenarios where $0.6\le {C}_{pop}<0.65$, the 95th percentile range of the bias was, $(-0.580, -0.007)$. The same trend was observed when ${Q}_{meas}$ increased. However, this is likely because under our data generating mechanism a larger ${Q}_{meas}$ on average results in a larger ${R}_{CS\_pop}^{2}$ and ${C}_{pop}$. This assumption is tested and verified under a different data generating mechanism where ${Q}_{meas}$ and the C-statistic are independent from each other which is detailed in “Impact of using a different data generating mechanism” section.

**Table 1:** Simulation study 1: The mean, and 2.5th, 25th, 50th, 75th and 97.5th percentiles of the bias of ${\widehat{\mathrm{S}}}_{\mathrm{VH}}$ and ${\widehat{\mathrm{S}}}_{\mathrm{boot}}$ across scenarios where data was simulated with a non-zero covariance structure, stratified by ${\mathrm{C}}_{\mathrm{pop}}$

	**2.5%**	**25%**	**50%**	**75%**	**97.5%**	**Mean (sd)**
All (14,156 scenarios)						
Magnitude of bias of ${\widehat{{\boldsymbol{S}}}}_{{\boldsymbol{V}}{\boldsymbol{H}}}$	0.001	0.011	0.032	0.097	0.384	0.077 (0.108)
Magnitude of bias of ${\widehat{{\boldsymbol{S}}}}_{{\boldsymbol{b}}{\boldsymbol{o}}{\boldsymbol{o}}{\boldsymbol{t}}}$	0.000	0.003	0.007	0.024	0.155	0.024 (0.040)
All (14,156 scenarios)						
Bias of ${\widehat{{\boldsymbol{S}}}}_{{\boldsymbol{V}}{\boldsymbol{H}}}$	−0.262	−0.006	0.014	0.060	0.323	0.026 (0.130)
Bias of ${\widehat{{\boldsymbol{S}}}}_{{\boldsymbol{b}}{\boldsymbol{o}}{\boldsymbol{o}}{\boldsymbol{t}}}$	−0.150	−0.003	0.003	0.013	0.082	0.001 (0.047)
$0.55\le {{\boldsymbol{C}}}_{{\boldsymbol{p}}{\boldsymbol{o}}{\boldsymbol{p}}}<0.6$ (306 scenarios)						
Bias of ${\widehat{{\boldsymbol{S}}}}_{{\boldsymbol{V}}{\boldsymbol{H}}}$	−0.780	−0.322	−0.150	−0.074	−0.027	−0.230 (0.218)
Bias of ${\widehat{{\boldsymbol{S}}}}_{{\boldsymbol{b}}{\boldsymbol{o}}{\boldsymbol{o}}{\boldsymbol{t}}}$	−0.110	−0.014	0.001	0.015	0.148	0.003 (0.078)
$0.6\le {{\boldsymbol{C}}}_{{\boldsymbol{p}}{\boldsymbol{o}}{\boldsymbol{p}}}<0.65$ (818 scenarios)						
Bias of ${\widehat{{\boldsymbol{S}}}}_{{\boldsymbol{V}}{\boldsymbol{H}}}$	−0.580	−0.208	−0.081	−0.035	−0.007	−0.144 (0.159)
Bias of ${\widehat{{\boldsymbol{S}}}}_{{\boldsymbol{b}}{\boldsymbol{o}}{\boldsymbol{o}}{\boldsymbol{t}}}$	−0.053	−0.005	0.005	0.022	0.161	0.017 (0.058)
$0.65\le {{\boldsymbol{C}}}_{{\boldsymbol{p}}{\boldsymbol{o}}{\boldsymbol{p}}}<0.7$ (1,469 scenarios)						
Bias of ${\widehat{{\boldsymbol{S}}}}_{{\boldsymbol{V}}{\boldsymbol{H}}}$	−0.358	−0.091	−0.034	−0.013	0.003	−0.073 (0.102)
Bias of ${\widehat{{\boldsymbol{S}}}}_{{\boldsymbol{b}}{\boldsymbol{o}}{\boldsymbol{o}}{\boldsymbol{t}}}$	−0.023	−0.002	0.006	0.028	0.128	0.020 (0.040)
$0.7\le {{\boldsymbol{C}}}_{{\boldsymbol{p}}{\boldsymbol{o}}{\boldsymbol{p}}}<0.75$ (2,488 scenarios)						
Bias of ${\widehat{{\boldsymbol{S}}}}_{{\boldsymbol{V}}{\boldsymbol{H}}}$	−0.129	−0.015	−0.002	0.008	0.089	−0.005 (0.049)
Bias of ${\widehat{{\boldsymbol{S}}}}_{{\boldsymbol{b}}{\boldsymbol{o}}{\boldsymbol{o}}{\boldsymbol{t}}}$	−0.011	0.000	0.007	0.031	0.091	0.018 (0.028)
$0.75\le {{\boldsymbol{C}}}_{{\boldsymbol{p}}{\boldsymbol{o}}{\boldsymbol{p}}}<0.8$ (4,140 scenarios)						
Bias of ${\widehat{{\boldsymbol{S}}}}_{{\boldsymbol{V}}{\boldsymbol{H}}}$	−0.010	0.007	0.020	0.067	0.246	0.049 (0.069)
Bias of ${\widehat{{\boldsymbol{S}}}}_{{\boldsymbol{b}}{\boldsymbol{o}}{\boldsymbol{o}}{\boldsymbol{t}}}$	−0.088	−0.001	0.005	0.017	0.049	0.005 (0.028)
$0.8\le {{\boldsymbol{C}}}_{{\boldsymbol{p}}{\boldsymbol{o}}{\boldsymbol{p}}}<0.85$ (4,295 scenarios)						
Bias of ${\widehat{{\boldsymbol{S}}}}_{{\boldsymbol{V}}{\boldsymbol{H}}}$	0.005	0.019	0.049	0.139	0.387	0.097 (0.107)
Bias of ${\widehat{{\boldsymbol{S}}}}_{{\boldsymbol{b}}{\boldsymbol{o}}{\boldsymbol{o}}{\boldsymbol{t}}}$	−0.174	−0.005	0.001	0.006	0.021	−0.018 (0.052)
$0.85\le {{\boldsymbol{C}}}_{{\boldsymbol{p}}{\boldsymbol{o}}{\boldsymbol{p}}}<0.9$ (598 scenarios)						
Bias of ${\widehat{{\boldsymbol{S}}}}_{{\boldsymbol{V}}{\boldsymbol{H}}}$	0.009	0.026	0.075	0.179	0.487	0.131 (0.141)
Bias of ${\widehat{{\boldsymbol{S}}}}_{{\boldsymbol{b}}{\boldsymbol{o}}{\boldsymbol{o}}{\boldsymbol{t}}}$	−0.204	−0.026	−0.002	0.002	0.010	−0.033 (0.064)

As the sample size N increases, the difference between $mean\left({\widehat{S}}_{VH}\right)$ and $mean({S}_{opt})$ gets smaller. The 95-percentile range of the bias is $(-0.390, 0.449)$ for scenarios with $100\le N<200$, and $(-0.060, 0.019)$ for scenarios with $\mathrm{2,500}\le N<\mathrm{5,000}$ (supplementary Table 1). Despite a reduction in the bias as $N$ increases, we still observe under-prediction or over-prediction over the same ranges of ${R}_{CS\_pop}^{2}$ and ${C}_{pop}$ (Fig. 1). The pattern for EPP is a mix between the plots for ${Q}_{meas}$ and $N$, to be expected given that EPP is a function of these two metrics.

**Fig. 2:**
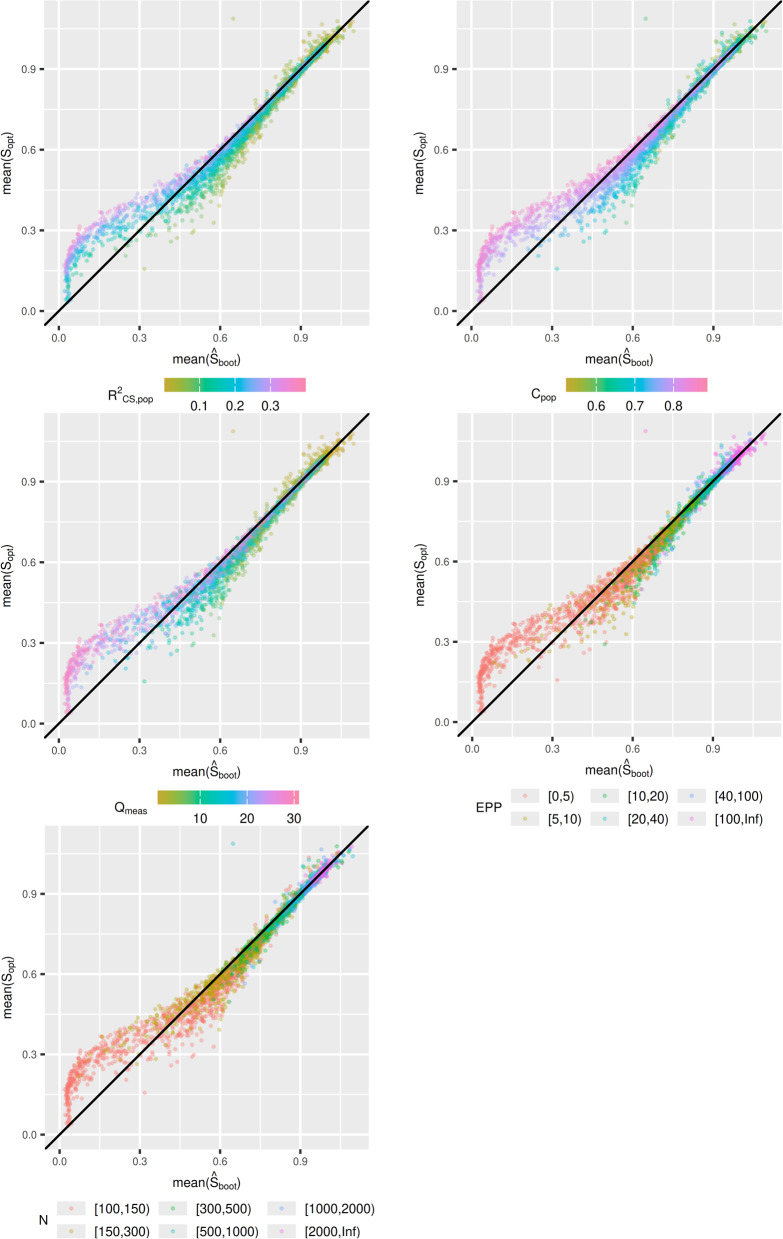
Simulation study 1: $\mathrm{mean}\left({\widehat{\mathrm{S}}}_{\mathrm{boot}}\right)$ plotted against $\mathrm{mean}({\mathrm{S}}_{\mathrm{opt}})$

Figure 2 contains $mean\left({\widehat{S}}_{boot}\right)$ plotted against $mean({S}_{opt})$. It can be seen that ${\widehat{S}}_{boot}$ is a far superior estimator than ${S}_{VH}$. This is supported by Table 1, as the mean magnitude of the bias across all scenarios is $0.024$ for ${\widehat{S}}_{boot}$, compared to $0.077$ for ${\widehat{S}}_{VH}$ ($0.007$ vs $0.032$ for median of the magnitude of the bias). The bias of ${\widehat{S}}_{boot}$ is generally quite small, especially when the true ${S}_{opt}$ is about 0.7 or above, and only when ${S}_{opt}$ is very small (< 0.3) does performance of ${\widehat{S}}_{boot}$ drop considerably. Sample size criteria can be used to protect against ending up in a scenario with ${S}_{opt}$ in this range. Furthermore, the bias of ${\widehat{S}}_{boot}$ is not as highly dependent on ${C}_{pop}$ as that of ${\widehat{S}}_{VH}$. While the 2.5th and 97.5th percentiles of bias decreases as ${C}_{pop}$ increases, the median remains close to $0$ across all values of ${C}_{pop}$ (Table 1). The scenarios skewing the 2.5th and 97.5th percentiles are when there is a small sample size, which has a differing effect depending on ${C}_{pop}$ (see Fig. 2). When the sample size is large, the impact of ${C}_{pop}$ greatly reduced, although there appears to be some over prediction for scenarios with lower ${C}_{pop}$.

Note that we only present the agreement at the mean level, the bias. At the individual model level, there is variability around the means, and this is often considerable especially in small development sample sizes, suggesting that – although it works well on average – there is no guarantee that even bootstrapping will perfectly address overfitting in a single study. This agrees with previous research [3, 5, 6], and we also highlight this using our simulated data in section ‘further results on the variance of the estimators’.

Table 2 contains the 50th, 90th and 99th percentiles (across the 14,161 different scenarios) of the Monte Carlo Standard Error (MCE) of the bias of ${\widehat{S}}_{VH}$ and ${\widehat{S}}_{boot}$. We see the MCE is below 0.007 (${\widehat{S}}_{VH}$) and 0.006 (${\widehat{S}}_{boot}$) in 50% of the scenarios, below 0.018 (${\widehat{S}}_{VH}$) and 0.012 (${\widehat{S}}_{boot}$) in 90% of scenarios, and below 0.065 (${\widehat{S}}_{VH}$) and 0.032 (${\widehat{S}}_{boot}$) in 99% of scenarios. The MCE increases as ${C}_{pop}$ decreases.

**Table 2:** Simulation study 1: 50th, 90th and 99th percentiles of the Monte Carlo Standard Error (MCE) of the bias of ${\widehat{\mathrm{S}}}_{\mathrm{VH}}$ and ${\widehat{\mathrm{S}}}_{\mathrm{boot}}$ across scenarios where data was simulated with a non-zero covariance structure, stratified by ${\mathrm{C}}_{\mathrm{pop}}$

	**50%**	**90%**	**99%**
All (14,161)			
MCE of bias of ${\widehat{S}}_{{\boldsymbol{V}}{\boldsymbol{H}}}$	0.007	0.018	0.065
MCE of bias of ${\widehat{{\boldsymbol{S}}}}_{{\boldsymbol{b}}{\boldsymbol{o}}{\boldsymbol{o}}{\boldsymbol{t}}}$	0.006	0.012	0.032
$0.6\le {{\boldsymbol{C}}}_{{\boldsymbol{p}}{\boldsymbol{o}}{\boldsymbol{p}}}<0.65$ (818 scenarios)			
MCE of bias of ${\widehat{{\boldsymbol{S}}}}_{{\boldsymbol{V}}{\boldsymbol{H}}}$	0.014	0.053	0.160
MCE of bias of ${\widehat{{\boldsymbol{S}}}}_{{\boldsymbol{b}}{\boldsymbol{o}}{\boldsymbol{o}}{\boldsymbol{t}}}$	0.012	0.025	0.142
$0.65\le {{\boldsymbol{C}}}_{{\boldsymbol{p}}{\boldsymbol{o}}{\boldsymbol{p}}}<0.7$ (1,469 scenarios)			
MCE of bias of ${\widehat{{\boldsymbol{S}}}}_{{\boldsymbol{V}}{\boldsymbol{H}}}$	0.010	0.033	0.086
MCE of bias of ${\widehat{{\boldsymbol{S}}}}_{{\boldsymbol{b}}{\boldsymbol{o}}{\boldsymbol{o}}{\boldsymbol{t}}}$	0.009	0.018	0.035
$0.7\le {{\boldsymbol{C}}}_{{\boldsymbol{p}}{\boldsymbol{o}}{\boldsymbol{p}}}<0.75$ (2,488 scenarios)			
MCE of bias of ${\widehat{{\boldsymbol{S}}}}_{{\boldsymbol{V}}{\boldsymbol{H}}}$	0.008	0.019	0.034
MCE of bias of ${\widehat{{\boldsymbol{S}}}}_{{\boldsymbol{b}}{\boldsymbol{o}}{\boldsymbol{o}}{\boldsymbol{t}}}$	0.007	0.013	0.019
$0.75\le {{\boldsymbol{C}}}_{{\boldsymbol{p}}{\boldsymbol{o}}{\boldsymbol{p}}}<0.8$ (4,140 scenarios)			
MCE of bias of ${\widehat{{\boldsymbol{S}}}}_{{\boldsymbol{V}}{\boldsymbol{H}}}$	0.006	0.014	0.021
MCE of bias of ${\widehat{{\boldsymbol{S}}}}_{{\boldsymbol{b}}{\boldsymbol{o}}{\boldsymbol{o}}{\boldsymbol{t}}}$	0.005	0.009	0.013
$0.8\le {{\boldsymbol{C}}}_{{\boldsymbol{p}}{\boldsymbol{o}}{\boldsymbol{p}}}<0.85$ (4,295 scenarios)			
MCE of bias of ${\widehat{{\boldsymbol{S}}}}_{{\boldsymbol{V}}{\boldsymbol{H}}}$	0.006	0.012	0.015
MCE of bias of ${\widehat{{\boldsymbol{S}}}}_{{\boldsymbol{b}}{\boldsymbol{o}}{\boldsymbol{o}}{\boldsymbol{t}}}$	0.005	0.007	0.009
$0.85\le {{\boldsymbol{C}}}_{{\boldsymbol{p}}{\boldsymbol{o}}{\boldsymbol{p}}}<0.9$ (598 scenarios)			
MCE of bias of ${\widehat{{\boldsymbol{S}}}}_{{\boldsymbol{V}}{\boldsymbol{H}}}$	0.005	0.012	0.015
MCE of bias of ${\widehat{{\boldsymbol{S}}}}_{{\boldsymbol{b}}{\boldsymbol{o}}{\boldsymbol{o}}{\boldsymbol{t}}}$	0.004	0.007	0.008

**Fig. 3:**
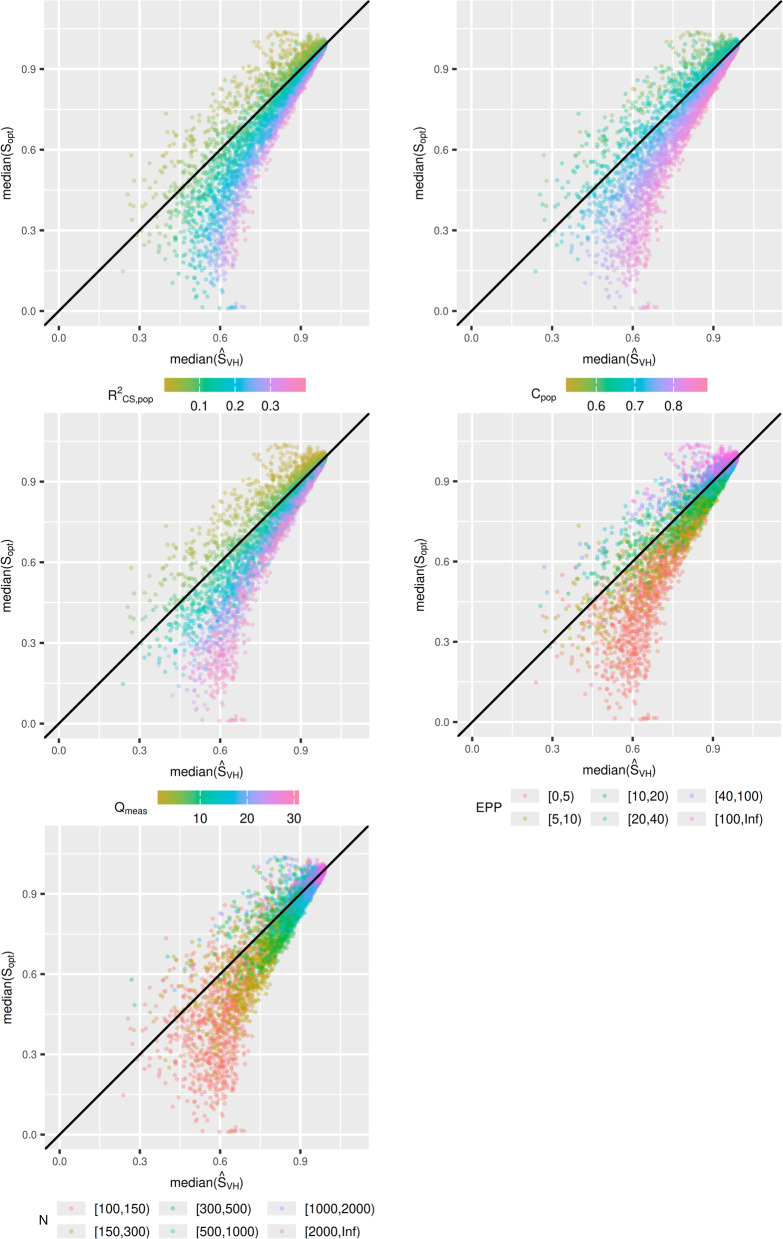
Simulation study 1: $\mathrm{median}\left({\widehat{\mathrm{S}}}_{\mathrm{VH}}\right)$ plotted against $\mathrm{median}\left({\mathrm{S}}_{\mathrm{opt}}\right)$

Figure 3 contains $median\left({\widehat{S}}_{VH}\right)$ plotted against $median({S}_{opt})$ for each scenario. The conclusions are broadly similar, however there are less scenarios where ${\widehat{S}}_{VH}$ drastically underpredicts ${S}_{opt}$, as the median is less susceptible to outliers. Looking at the data reveals that scenarios with this property have small sample sizes ($< 500$), only two or three measured predictors, and $>20$ unmeasured predictors. These are extremely high noise scenarios and are extreme edge cases in our parameter space. There is minimal difference between the mean and median plots for ${\widehat{S}}_{boot}$ (see supplementary material), indicating ${\widehat{S}}_{boot}$ results in less extreme outlier estimates of ${S}_{opt}$.

### Further results on the variance of the estimators

**Fig. 4:**
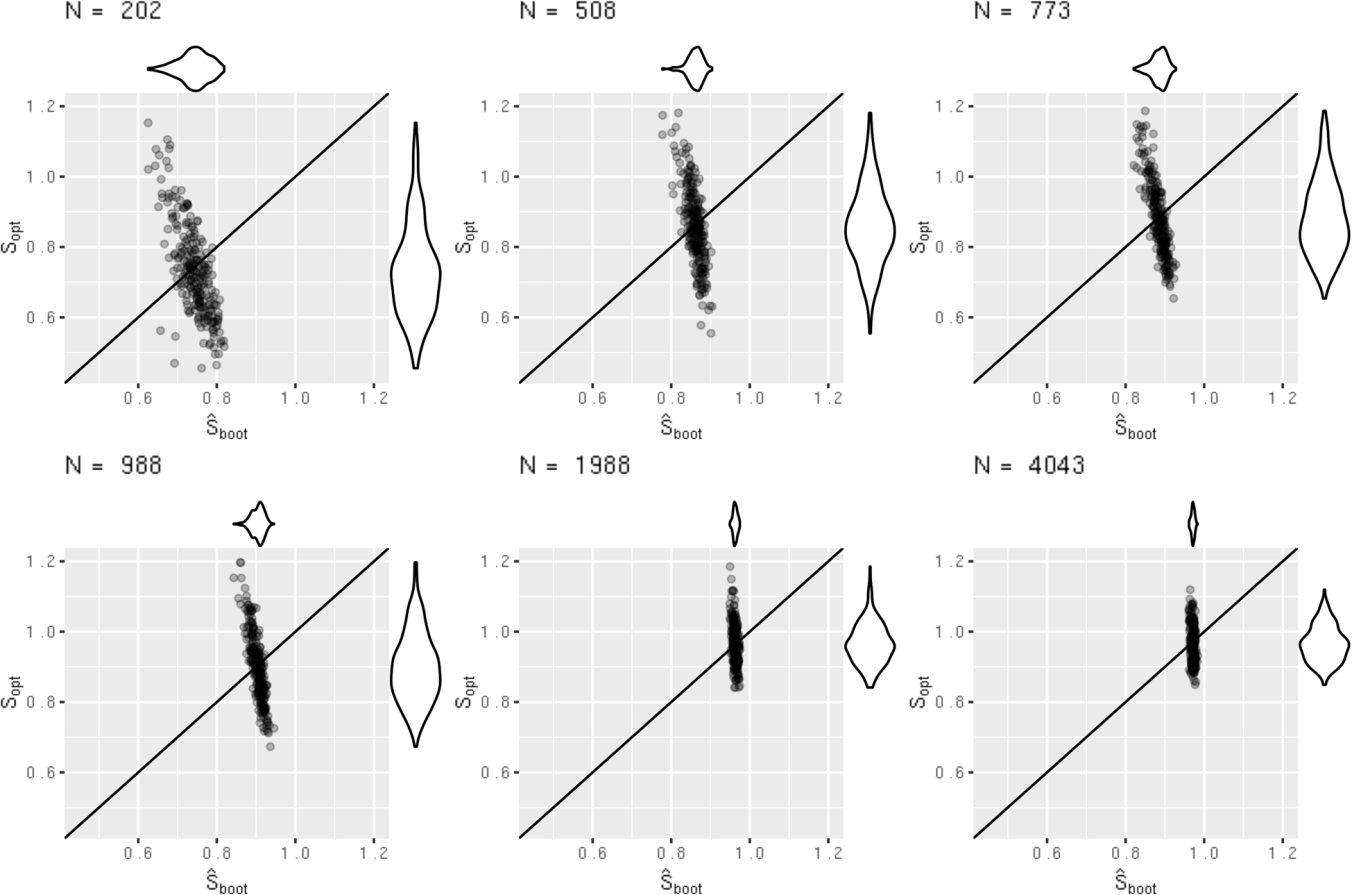
Instability plots, ${\mathrm{S}}_{\mathrm{opt}}$ plotted against ${\widehat{\mathrm{S}}}_{\mathrm{boot}}$ across 250 iterations in 6 scenarios from simulation study 1

Given the strong performance of bootstrapping in the simulation, it is important to also reiterate that bootstrapping is not a panacea, and our findings only indicate that it performs well on average. There is instability in this estimator and in any given study, with one sample of data, ${\widehat{S}}_{boot}$ will not necessarily estimate ${S}_{opt}$ correctly. We highlight this by picking out 6 of the scenarios from the simulation study 1 and plotting ${S}_{opt}$ against ${\widehat{S}}_{boot}$ for the 250 simulation iterations. We choose scenarios with sample sizes as close as possible to $250, 500, 750, 1000, 2000$ and $4000$ and where the difference between $mean({\widehat{S}}_{boot})$ and $mean({S}_{opt})$ was $<0.01$; i.e. ${\widehat{S}}_{boot}$ estimated ${S}_{opt}$ well on average in that scerario. For consistency across the plots, we chose scenarios with ${Q}_{meas}=10$. The plots are presented in Fig. 4.

We see that there is less variability in the estimator ${\widehat{S}}_{boot}$ compared to the amount of required shrinkage ${S}_{opt}$. This means in any given study, ${\widehat{S}}_{boot}$ will not necessarily correctly estimate the required amount of shrinkage. As sample size increases, the amount of variability in both ${\widehat{S}}_{boot}$ and ${S}_{opt}$ reduces. As all the values become closer to 1 this becomes less of an issue, however, the same behaviour of ${\widehat{S}}_{boot}$ is still observed. The standard deviation of estimators ${\widehat{S}}_{VH}$ and ${\widehat{S}}_{boot}$ are plotted against the standard deviation of ${S}_{opt}$ across all scenarios in the supplementary Figures S21—S24. The plot for ${\widehat{S}}_{boot}$ shows that the variability in ${\widehat{S}}_{boot}$ is often much lower than that of ${S}_{opt}$, supporting the results from Fig. 4. This supports existing research which shows that applying shrinkage techniques will not guarantee improved model performance [3, 5, 6].

## Simulation Study 2: Example performance of the Riley et al. [17] sample size criteria using original or Pavlou adjustment [19]

Our second simulation also follows the aims, data generating mechanisms, estimands, methods, performance metrics (ADEMP) [21] structure.

### Aims

The aim is to assess whether the amount of targeted shrinkage via the Riley sample size criterion 1 (calibration slope target of 0.9) is achieved (on average) when the model is applied in a large validation study. Specifically, we examine $mean\left({S}_{opt}\right)$ when using a development size of ${N=N}_{original}$ (criterion 1 of Riley’s proposal [17], based on ${\widehat{S}}_{VH}$) and ${N=N}_{sim}$ (based on an simulation-based adaptation of Pavlou et al [19]). This simulation study will act as a replication study [25]. The key concepts around defining a sample size for developing a prediction model based on the heuristic shrinkage factor and on the simulation-based approach by Pavlou et al. [19]

### Key concepts when estimating a sample size to target a pre-specified level of global shrinkage

Among other criteria, Riley et al.,[17] proposed choosing a sample size $N$ that satisfies: 

4 $$N=\frac{p}{\left(S-1\right)*ln\left(1-\frac{{R}_{CS\_adj}^{2}}{S}\right)}$$

where $p$ is the number of predictor parameters prior to variable selection, ${R}_{CS\_adj}^{2}$ is the expected Cox-Snell [22] ${R}^{2}$ of the model, and ${S}$ is the desired level of shrinkage. This approach targets the heuristic shrinkage factor of Van Houwelingen and Le Cessie [7] (${\widehat{S}}_{VH}$) to be at the desired level. This sample size formula has an analytical solution.

Pavlou et al.,[19] proposed using a simulation based approach. The anticipated outcome prevalence, the expected C-statistic of the model, desired shrinkage and number of predictor parameters must be pre-specified. With some extra assumptions on the distribution, data can then be simulated which have the corresponding number of predictor parameters, outcome prevalence, C-statistic. Development and validation cohorts are then simulated in an iterative manner fitting models to estimate expected shrinkage. This process is repeated until a development sample size is reached, where the expected shrinkage is sufficiently close to the target shrinkage.

### Data generating mechanisms

The data generation process and creation of scenarios is identical to that described in “Data generating mechanisms” section. However, the process for the simulation is different, as follows. Input parameters ${Q}_{meas}, {Q}_{unmeas}, \beta$ and $\Omega$ were simulated for 25,000 scenarios (half of which had $\Omega$ as the identity matrix, inducing no correlation between the predictors). For each scenario the simulation was performed as follows:

- Step 1 – step 3 from simulation study 1.
- Step 4: Calculate ${N}_{original}$ and ${N}_{sim}$ to target a global shrinkage factor of 0.9, using ${R}_{CS\_pop}^{2}$ and ${C}_{pop}$ as the expected model performance.

Repeat the following steps 250 times:

- Step 5: Simulate datasets of size ${N}_{original}$ and ${N}_{sim}$.
- Step 6: Develop binary logistic regression models in the two development datasets, using the first ${Q}_{meas}$ variables as predictors.
- Step 7: Generate estimated risks for individuals in the validation cohort, $\widehat{p}$, using the models developed in step 6.
- Step 8: Calculate ${S}_{opt}$ by calculating the calibration slope in the large validation cohort for each developed model.

### Estimands, methods and performance metric

We assess whether the mean calibration slope of models developed in datasets of size ${N}_{original}$ and ${N}_{sim}$ is at the desired threshold of 0.9. The performance metric of interest is therefore $mean\left({S}_{opt,k}\right)$ in each scenario $k$, where the mean is calculated across the 250 simulation iterations in each scenario. We report the percentile range in $mean({S}_{opt,k})$ across all the scenarios, and subgroups defined by ${C}_{pop}$. The bias in scenario $k$ is the mean difference between ${S}_{opt}$ and the targeted level of shrinkage:

$${bias}_{k}=\frac{\sum_{m=1}^{250}\left({S}_{opt,k,m}-0.9\right)}{250}.$$

We also present the mean of the bias, and magnitude of the bias, across all scenarios, and scenarios stratified by ${C}_{pop}$.

## Results

Table 3 contains the 2.5th, 25th, 50th, 75th and 97.5th of $mean\left({S}_{opt}\right)$, when models are developed on datasets with sample sizes meeting criteria of Riley et al., [17] (${N}_{original}$) and the adjustment of Pavlou et al., [19] (${N}_{sim}$) targeting a shrinkage factor of 0.9. These scenarios had non-zero covariance between predictor variables. It can be seen that:

- The mean magnitude of the bias of ${S}_{opt}$ across all scenarios was 0.041 when models were developed on datasets with sample size ${N}_{original}$, compared to 0.004 for ${N}_{sim}$.
- For ${C}_{pop}>0.7$, ${N}_{original}$ lead to an ${S}_{opt}$ lower than the target of 0.9 on average. For ${C}_{pop}<0.7$, ${N}_{original}$ lead to an ${S}_{opt}$ higher than the target of 0.9 on average. ${N}_{sim}$ resulted in an average ${S}_{opt}$ close to 0.9, irrespective of the value of ${C}_{pop}$.
- The $mean\left({S}_{opt}\right)$ has a 95 percentile range of $0.814-0.985$ when models are developed on datasets with sample size ${N}_{original}$, compared to $0.882-0.921$ for ${N}_{sim}$. Therefore, the variability in ${S}_{opt}$ is larger when using ${N}_{original}$ than ${N}_{sim}$, although this is expected due to the latter generally being larger.

**Table 3:** Simulation study 2: The 2.5th, 25th, 50th, 75th and 97.5th percentiles of $\mathrm{mean}\left({\mathrm{S}}_{\mathrm{opt}}\right)$ across scenarios where data was simulated with a non-zero covariance structure, stratified by ${\mathrm{C}}_{\mathrm{pop}}$

	**2.5%**	**25%**	**50%**	**75%**	**97.5%**	**Mean bias (sd)**	**Mean magnitude of bias (sd)**
**Scenarios where **${{\boldsymbol{N}}}_{{\boldsymbol{s}}{\boldsymbol{i}}{\boldsymbol{m}}}$** converged (11,944 scenarios)**							
${{\boldsymbol{N}}}_{{\boldsymbol{o}}{\boldsymbol{r}}{\boldsymbol{i}}{\boldsymbol{g}}{\boldsymbol{i}}{\boldsymbol{n}}{\boldsymbol{a}}{\boldsymbol{l}}}$	0.814	0.848	0.869	0.896	0.985	−0.023 (0.049)	0.041 (0.035)
${{\boldsymbol{N}}}_{{\boldsymbol{s}}{\boldsymbol{i}}{\boldsymbol{m}}}$	0.882	0.896	0.900	0.905	0.921	0.000 (0.043)	0.004 (0.042)
$0.55\le {{\boldsymbol{C}}}_{{\boldsymbol{p}}{\boldsymbol{o}}{\boldsymbol{p}}}<0.6$** (365 scenarios)**							
${{\boldsymbol{N}}}_{{\boldsymbol{o}}{\boldsymbol{r}}{\boldsymbol{i}}{\boldsymbol{g}}{\boldsymbol{i}}{\boldsymbol{n}}{\boldsymbol{a}}{\boldsymbol{l}}}$	0.917	0.944	0.972	1.000	1.238	0.093 (0.098)	0.093 (0.098)
${{\boldsymbol{N}}}_{{\boldsymbol{s}}{\boldsymbol{i}}{\boldsymbol{m}}}$	0.788	0.886	0.903	0.928	1.494	0.003 (0.177)	0.019 (0.166)
$0.6\le {{\boldsymbol{C}}}_{{\boldsymbol{p}}{\boldsymbol{o}}{\boldsymbol{p}}}<0.65$** (694 scenarios)**							
${{\boldsymbol{N}}}_{{\boldsymbol{o}}{\boldsymbol{r}}{\boldsymbol{i}}{\boldsymbol{g}}{\boldsymbol{i}}{\boldsymbol{n}}{\boldsymbol{a}}{\boldsymbol{l}}}$	0.904	0.920	0.935	0.959	1.113	0.048 (0.055)	0.049 (0.054)
${{\boldsymbol{N}}}_{{\boldsymbol{s}}{\boldsymbol{i}}{\boldsymbol{m}}}$	0.827	0.892	0.902	0.913	1.047	0.002 (0.081)	0.010 (0.076)
$0.65\le {{\boldsymbol{C}}}_{{\boldsymbol{p}}{\boldsymbol{o}}{\boldsymbol{p}}}<0.7$** (1,271 scenarios)**							
${{\boldsymbol{N}}}_{{\boldsymbol{o}}{\boldsymbol{r}}{\boldsymbol{i}}{\boldsymbol{g}}{\boldsymbol{i}}{\boldsymbol{n}}{\boldsymbol{a}}{\boldsymbol{l}}}$	0.888	0.903	0.913	0.926	0.981	0.018 (0.025)	0.020 (0.023)
${{\boldsymbol{N}}}_{{\boldsymbol{s}}{\boldsymbol{i}}{\boldsymbol{m}}}$	0.872	0.894	0.901	0.908	0.925	0.001 (0.019)	0.007 (0.016)
$0.7\le {{\boldsymbol{C}}}_{{\boldsymbol{p}}{\boldsymbol{o}}{\boldsymbol{p}}}<0.75$** (2,102 scenarios)**							
${{\boldsymbol{N}}}_{{\boldsymbol{o}}{\boldsymbol{r}}{\boldsymbol{i}}{\boldsymbol{g}}{\boldsymbol{i}}{\boldsymbol{n}}{\boldsymbol{a}}{\boldsymbol{l}}}$	0.871	0.882	0.890	0.900	0.931	−0.007 (0.016)	0.014 (0.011)
${{\boldsymbol{N}}}_{{\boldsymbol{s}}{\boldsymbol{i}}{\boldsymbol{m}}}$	0.885	0.895	0.901	0.905	0.917	0.001 (0.010)	0.005 (0.008)
$0.75\le {{\boldsymbol{C}}}_{{\boldsymbol{p}}{\boldsymbol{o}}{\boldsymbol{p}}}<0.8$** (3,417 scenarios)**							
${{\boldsymbol{N}}}_{{\boldsymbol{o}}{\boldsymbol{r}}{\boldsymbol{i}}{\boldsymbol{g}}{\boldsymbol{i}}{\boldsymbol{n}}{\boldsymbol{a}}{\boldsymbol{l}}}$	0.848	0.859	0.867	0.875	0.893	−0.032 (0.012)	0.032 (0.011)
${{\boldsymbol{N}}}_{{\boldsymbol{s}}{\boldsymbol{i}}{\boldsymbol{m}}}$	0.888	0.896	0.900	0.904	0.912	−0.000 (0.006)	0.004 (0.004)
$0.8\le {{\boldsymbol{C}}}_{{\boldsymbol{p}}{\boldsymbol{o}}{\boldsymbol{p}}}<0.85$** (3,471 scenarios)**							
${{\boldsymbol{N}}}_{{\boldsymbol{o}}{\boldsymbol{r}}{\boldsymbol{i}}{\boldsymbol{g}}{\boldsymbol{i}}{\boldsymbol{n}}{\boldsymbol{a}}{\boldsymbol{l}}}$	0.818	0.834	0.843	0.851	0.867	−0.057 (0.012)	0.057 (0.012)
${{\boldsymbol{N}}}_{{\boldsymbol{s}}{\boldsymbol{i}}{\boldsymbol{m}}}$	0.890	0.897	0.900	0.904	0.910	0.000 (0.005)	0.003 (0.003)
$0.85\le {{\boldsymbol{C}}}_{{\boldsymbol{p}}{\boldsymbol{o}}{\boldsymbol{p}}}<0.9$** (564 scenarios)**							
${{\boldsymbol{N}}}_{{\boldsymbol{o}}{\boldsymbol{r}}{\boldsymbol{i}}{\boldsymbol{g}}{\boldsymbol{i}}{\boldsymbol{n}}{\boldsymbol{a}}{\boldsymbol{l}}}$	0.784	0.807	0.815	0.822	0.838	−0.086 (0.013)	0.086 (0.013)
${{\boldsymbol{N}}}_{{\boldsymbol{s}}{\boldsymbol{i}}{\boldsymbol{m}}}$	0.891	0.897	0.901	0.904	0.909	0.001 (0.005)	0.003 (0.003)
**Scenarios where **${{\boldsymbol{N}}}_{{\boldsymbol{s}}{\boldsymbol{i}}{\boldsymbol{m}}}$** did not converge (235 scenarios)**							
${{\boldsymbol{N}}}_{{\boldsymbol{o}}{\boldsymbol{r}}{\boldsymbol{i}}{\boldsymbol{g}}{\boldsymbol{i}}{\boldsymbol{n}}{\boldsymbol{a}}{\boldsymbol{l}}}$	0.806	1.036	1.140	1.184	1.416	0.225 (0.161)	0.237 (0.144)
${{\boldsymbol{N}}}_{{\boldsymbol{s}}{\boldsymbol{i}}{\boldsymbol{m}}}$	NA	NA	NA	NA	NA	NA	NA

The 95-percentile range of $mean\left({S}_{opt}\right)$ for ${N}_{original}$ was sensitive to ${C}_{pop}$. As the C-statistic increased, the amount of shrinkage required became larger (smaller ${S}_{opt}$). The mean bias ranged from $0.093$ ($0.55\le {C}_{pop}<0.6$) to $-0.086$ ($0.85\le {C}_{pop}<0.9$). For ${N}_{sim}$, the 95-percentile range of $mean\left({S}_{opt}\right)$ was comparatively insensitive to changes in the C-statistic. The mean bias ranged from $0.003$ ($0.55\le {C}_{pop}<0.6$) to $0.001$ ($0.85\le {C}_{pop}<0.9$). ${N}_{sim}$ did not converge for a small number of scenarios (2%), which were predominately those with ${C}_{pop}<0.65$. The performance of ${N}_{original}$ in these scenarios is presented in the supplementary material. Scenarios were removed if $mean\left({S}_{opt}\right)$ was either negative or $>2$, which happened 72 times under ${N}_{sim}$ and 9 times under ${N}_{original}$. These were all scenarios where ${C}_{pop}<0.6$ and there was one predictor parameter, so caution may be needed in this setting. A scatter plot showing the relationship between ${N}_{original}$ and ${N}_{sim}$ is presented in the supplementary Figure S25. There is a positive correlation, but it can also be observed that ${N}_{sim}$ mostly higher than ${N}_{original}$, except when ${C}_{pop}$ is low.

###  Further results on the variance of ${S}_{opt}$ when sample size criteria are met

**Fig. 5:**
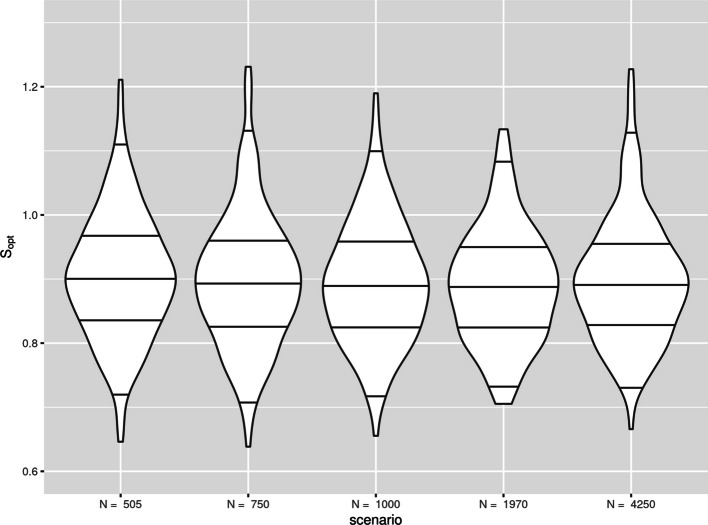
Instability plots of ${\mathrm{S}}_{\mathrm{opt}}$ across 250 iterations in 6 scenarios from simulation study 2 with sample size ${\mathrm{N}}_{\mathrm{sim}}$ targeting a shrinkage of 0.9. Horizontal lines at 2.5th, 25th, 50th, 75th and 97.5th percentiles

Given the strong performance of ${{\boldsymbol{N}}}_{{\boldsymbol{s}}{\boldsymbol{i}}{\boldsymbol{m}}}$ in the simulation, it is important to reiterate that our findings only indicate that it performs well on average. While we found ${N}_{sim}$ resulted in $mean({S}_{opt})\ge 0.9$ in most scenarios, there is still considerable variation in ${S}_{opt}$ across each simulation iteration within a scenario. In Fig. 5 we present data from 6 scenarios from simulation study 2, with sample size ${N}_{sim}$ as close to $250, 500, 750, 1000, 2000$ and $4000$ as possible, with ${Q}_{meas}=10$. Note, ${N}_{sim}$ was always targeting an ${S}_{opt}$ of 0.9. We see considerable variation in performance across simulation iterations. In practical terms, this means that working with a cohort of size ${N}_{sim}$ won’t ensure that ${S}_{opt}\ge 0.9$ after developing your model. It will only ensure that if you were to repeat the data collection and model development process numerous times, that $mean({S}_{opt})\ge 0.9$. This emphasises that the sample size criteria are indeed, the absolute minimum requirements, and one should always strive for a sample size much bigger. An equivalent plot for scenarios with ${N}_{original}$ at these sample sizes is presented in the supplementary Figure S26. Given the sample sizes are similar, there is no difference in the variability.

## Impact of using a different data generating mechanism

Using the data generating mechanism described in “Data generating mechanisms” section induced a strong association between the number of predictor parameters (${Q}_{meas}$) and the ${R}_{CS}^{2}$ and C-statistic metrics. This is because coefficients were simulated at random from a $Uniform\left(-\mathrm{0.5,0.5}\right)$ distribution, meaning increasing ${Q}_{meas}$ on average resulted in an increase in the C-statistic. In real world settings, you would expect this property, however, one would not necessarily expect the level of association to match that from these simulations. More importantly, when trying to interpret the results, given the relationship it’s difficult to tell whether it’s ${Q}_{meas}$, or ${R}_{CS}^{2}$ and the C-statistic which is driving the direction of the bias in ${\widehat{S}}_{VH}$ and ${\widehat{S}}_{boot}$. Given that ${Q}_{meas}$ is part of the formula for ${\widehat{S}}_{VH}$, this is an important question to answer. Therefore, the simulations were repeated using a data generating mechanism which detaches ${R}_{CS}^{2}$ and the C-statistic from ${Q}_{meas}$.

In this alternative data generating mechanism, ${Q}_{meas}$ was simulated at random uniformly as a random integer between $1$ and $30$, however there were no unmeasured predictors simulated (${Q}_{unmeas}=0$). The target C-statistic was simulated at random from a $Uniform\left(\mathrm{0.55,0.95}\right)$ distribution. Initial coefficients were simulated according to a to ${\beta }_{j}\sim Uniform\left(-\mathrm{0.5,0.5}\right) \forall j$ distribution, but were then rescaled using the methods of Austin [26] to result in a model with the target C-statistic. Under this data generating mechanism, ${Q}_{meas}$ and the C-statistic were independent from each other. All other aspects of the simulation studies remained the same. All results are presented in supplementary material.

**Fig. 6:**
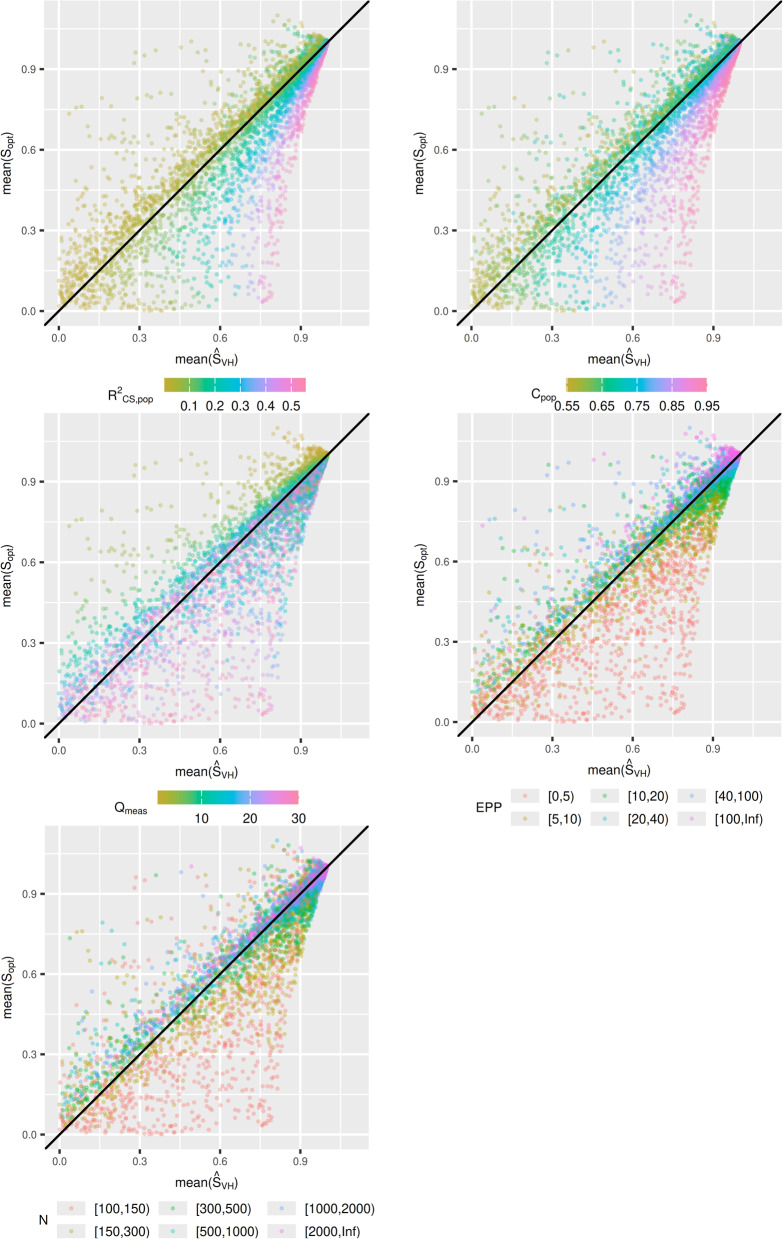
Simulation study 1 under data generating where ${\mathrm{Q}}_{\mathrm{meas}}$ and C-statistic are independent: $\mathrm{mean}\left({\widehat{\mathrm{S}}}_{\mathrm{VH}}\right)$ plotted against $\mathrm{mean}({\widehat{\mathrm{S}}}_{\mathrm{opt}})$

**Fig. 7:**
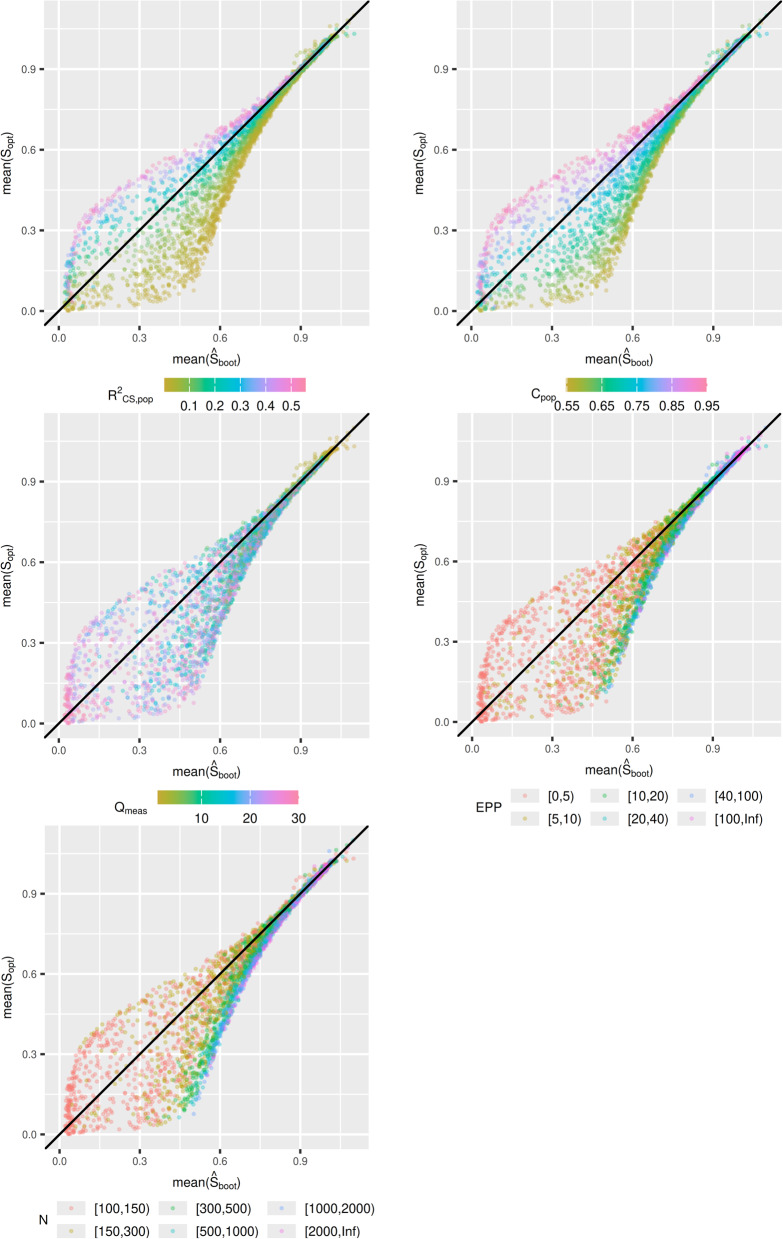
Simulation study 1 under data generating where ${\mathrm{Q}}_{\mathrm{meas}}$ and C-statistic are independent: $\mathrm{mean}\left({\widehat{\mathrm{S}}}_{\mathrm{boot}}\right)$ plotted against $\mathrm{mean}({\widehat{\mathrm{S}}}_{\mathrm{opt}})$

The bias of ${\widehat{S}}_{VH}$ and ${\widehat{S}}_{boot}$ from simulation study 1 under this new data generating mechanism are presented in Figs. 6 and 7 respectively. It is clear from Fig. 6 that the bias in ${\widehat{S}}_{VH}$ is strongly related to ${R}_{CS}^{2}$ and the C-statistic, rather than ${Q}_{meas}$. The relationship between ${Q}_{meas}$ and the bias is difficult to discern. For very low ${Q}_{meas}$ (< 3), it appears that ${S}_{VH}$ tends to underestimate ${S}_{opt}$, but this is not always the case. For other values of ${Q}_{meas}$, ${\widehat{S}}_{VH}$ might under-predict slightly, predict well, or over-predict possibly by a large amount.

Figure 7 indicates that there is a strong relationship between either the ${R}_{CS}^{2}$ or C-statistic, and the direction of the bias of ${\widehat{S}}_{boot},$ which may not have been obvious from Fig. 2. When ${R}_{CS}^{2}$ and the C-statistic are high, ${\widehat{S}}_{boot}$ will underestimate ${S}_{opt},$ and for lower values ${S}_{boot}$ will overestimate${S}_{opt}$. As the amount of required shrinkage is decreases (${S}_{opt}$ increases), the magnitude of this bias decreases. For mean$({S}_{opt})>0.7$, ${S}_{boot}$ performs well, and for mean$({S}_{opt})>0.9$, ${S}_{boot}$ performs very well. The results for simulation study 2 under the new data generating mechanism were consistent with the previous data generating mechanism and led to no new conclusions.

To conclude, the results from these supplementary analyses show that while simulation study 1 indicated that introducing more predictors into a model may result in ${\widehat{S}}_{VH}$ over-predicting ${S}_{opt}$, this is only true if these variables have predictive value and result in an increase of the actual ${R}_{CS}^{2}$ and the C-statistic. Figure 7 also revealed that there is a relationship between ${R}_{CS}^{2}$ and the C-statistic with the direction of bias of ${\widehat{S}}_{boot}$, although the magnitude of this bias is very small unless a large amount of shrinkage is required.

## Examples to demonstrate impact of findings

### Example 1: Impact on developing a model using a global shrinkage factor

We now highlight the difference between the heuristic shrinkage factor and bootstrapped shrinkage factor in a clinical example predicting AKI in the first 24-h after admission to an ICU. This examples utilises the MIMIC-III dataset, which contains freely available and de-identified critical care data from the Beth Israel Deaconess Medical Center in Boston, Massachusetts, between 2001 and 2012 [27]. Inclusion criteria were over 18 years of age, with a minimum length of stay of 24 h on ICU, and each patient’s first admission; we excluded anyone with a baseline eGFR < 60 mL/min/1.73m2. This resulted in a cohort of N = 20,413 individuals. There were 3,528 events (outcome proportion 17.28%).

We considered the development of an unpenalised logistic regression model with either 10 or 20 predictors ($P$), in a dataset of size 100, 250, 500, 1000. For every sample size and number of predictor parameters, we sampled 1000 datasets (without replacement) at random from the cohort of individuals meeting our inclusion criteria. Note that this is not big enough to replicate the process of sampling from the population, and therefore we could not estimate ${S}_{opt}$. Instead, for each iteration we calculated ${\widehat{S}}_{VH}$ and ${\widehat{S}}_{boot}$ (with 200 bootstrap samples). We present the 2.5th, 25th, 50th, 75th and 97.5th percentiles of the difference between ${\widehat{S}}_{VH}$ and ${\widehat{S}}_{boot}$, as well as the mean of each estimator (Table 4). We are also unable to estimate ${C}_{pop}$; we can only calculate the C-statistics of models developed in the entire cohort, $0.69$ ($P=10$) and $0.70$ ($P=20$). We are also unable to calculate ${S}_{opt}$ given the size of the dataset, but present the shrinkage required when validating the model in the full dataset of size N = 20,413 ($S$).

**Table 4:** The 2.5th, 25th, 50th, 75th and 97.5th percentiles of the difference between ${\widehat{\mathrm{S}}}_{\mathrm{boot}}$ and ${\widehat{\mathrm{S}}}_{\mathrm{VH}}$ across the 500 randomly sampled datasets and developed models, mean $\left({\widehat{S}}_{boot}\right)$ and mean $\left({\widehat{S}}_{\mathrm{VH}}\right)$ and mean (S)

	**2.5%**	**25%**	**50%**	**75%**	**97.5%**	${\boldsymbol{m}}{\boldsymbol{e}}{\boldsymbol{a}}{\boldsymbol{n}}({\widehat{{\boldsymbol{S}}}}_{{\boldsymbol{V}}{\boldsymbol{H}}})$	${\boldsymbol{m}}{\boldsymbol{e}}{\boldsymbol{a}}{\boldsymbol{n}}({\widehat{{\boldsymbol{S}}}}_{{\boldsymbol{b}}{\boldsymbol{o}}{\boldsymbol{o}}{\boldsymbol{t}}})$	${\boldsymbol{m}}{\boldsymbol{e}}{\boldsymbol{a}}{\boldsymbol{n}}({\boldsymbol{S}})$
**10 predictors**								
N = 100	−0.216	−0.028	0.087	0.274	1.007	0.254	0.425	0.286
N = 250	−0.035	0.033	0.086	0.155	0.466	0.540	0.660	0.564
N = 500	−0.005	0.028	0.052	0.082	0.188	0.721	0.784	0.750
N = 1000	0	0.015	0.025	0.037	0.067	0.848	0.875	0.870
**20 predictors**								
N = 100	−0.651	−0.38	−0.212	−0.048	0.445	0.3	0.11	0.102
N = 250	−0.122	−0.031	0.035	0.109	0.37	0.447	0.504	0.359
N = 500	−0.048	0	0.03	0.074	0.189	0.619	0.662	0.570
N = 1000	−0.027	−0.001	0.012	0.027	0.067	0.77	0.784	0.756

**Fig. 8:**
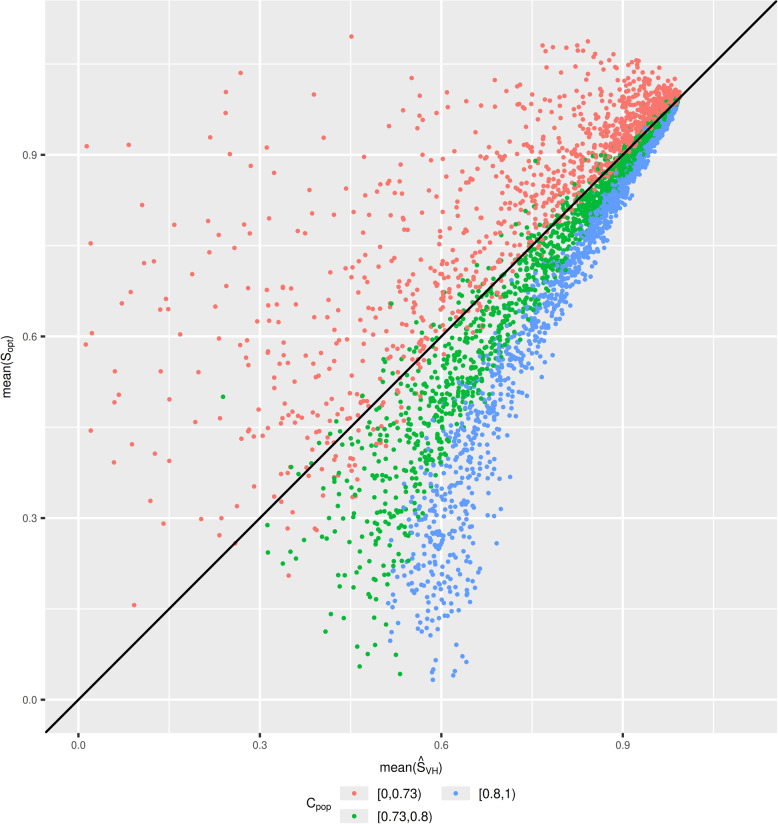
Simulation study 1: $\mathrm{mean}\left({\widehat{\mathrm{S}}}_{\mathrm{VH}}\right)$ plotted against $\mathrm{mean}({\widehat{\mathrm{S}}}_{\mathrm{opt}})$, presented with respect to ${\mathrm{C}}_{\mathrm{pop}}$ in a categorical manner. This allows us to be more precise at the threshold for which ${\mathrm{S}}_{\mathrm{VH}}$ over or under-estimates ${\mathrm{S}}_{\mathrm{opt}}$

Table 4 shows that when sample sizes are $<500$ there are considerable differences between ${\widehat{S}}_{VH}$ and ${\widehat{S}}_{boot}$. The findings from simulation study 1 therefore indicate that ${\widehat{S}}_{VH}$ is likely to be too low and be underestimating ${S}_{opt}$ in most of these scenarios. Figure 8 indicates that below the threshold of ${C}_{pop}=0.73$, ${\widehat{S}}_{VH}$ begins to underestimate ${S}_{opt}$ (see “Simulation study 2 (compare performance of sample size criteria)” section for more discussion of this). The estimates of ${C}_{pop}$ are likely to be slightly optimistic, and the true value of ${C}_{pop}$ to be lower than these, indicating ${\widehat{S}}_{VH}$ is underestimating ${S}_{opt}$. This is supported by the fact that $mean({\widehat{S}}_{VH})$ is smaller than $mean({\widehat{S}}_{boot})$ in most scenarios. On the contrary, the results from the simulation with the alternative data generating mechanism indicate that ${\widehat{S}}_{boot}$ is likely to be over estimating ${S}_{opt}$. The only scenario where this pattern does not occur is when $N = 100$ and $P = 20$. Here we find that $mean\left({\widehat{S}}_{VH}\right)=0.3$ and $mean\left({\widehat{S}}_{boot}\right)=0.11$. Developing a model with $20$ predictors in a cohort of size $100$ is likely to lead to large amount of overfitting, and subsequently require a lot of shrinkage (a small ${S}_{opt}$ value). At this point our simulation indicates both estimators could be performing very poorly, but model development would not be recommended anyway.

### Impact on sample size calculation

In recent years there has been widespread uptake of sample size formula for clinical prediction models [17, 28], which is hugely positive. Simulation study 2 supports the use of the simulation-based adaptation (${N}_{sim}$) suggested by Pavlou et al. [19]. We illustrate this in an applied example. Consider applied example 1 from the guidance article on sample size calculations in the BMJ [28]. The example considers a model predicting pre-eclampsia in pregnant women based on 13 predictors measured at 15 weeks gestation [29]. The outcome proportion was 0.05 and the C-statistic was 0.71.

Suppose we want to replicate this model in a new population and need to derive a minimum sample size. If we assume the same C-statistic will be achieved in this new population, then this corresponds to an ${R}_{CS\_adj}^{2}$of about 0.028 [30]. For this, the minimum required sample size to target a heuristic shrinkage factor of 0.9 can be calculated using *pmsampsize* [30], to give ${N}_{original}=4114$. In contrast, the simulation-based approach using the R package *samplesizedev* [19], suggests ${N}_{sim}=3820$. The required sample sizes are therefore quite similar, although the minimum required sample size according to the simulation-based approach is slightly less than the approach targeting the heuristic shrinkage factor. This agreement makes sense, given that simulation study 2 indicated both ${N}_{original}$ and ${N}_{sim}$ result in the desired level of shrinkage (on average) for a C-statistic of 0.71.

Now consider two other scenarios. In the first there are 10 extra predictors available in the data representing this new population, and inclusion of these predictors is assumed to increase the C-statistic of the model up to 0.85. If we repeat the sample size calculations using $p=23$ and $C=0.85$, we find that ${N}_{original}=2157$ and ${N}_{sim}=2985$. The minimum required sample size from the simulation-based approach is now considerably bigger (38%) than the approach targeting the heuristic shrinkage factor. This is driven by the larger C-statistic, and agrees with our simulation study results which suggest a larger sample size than ${N}_{original}$ is needed to target in that situation.

Now consider a scenario where only 5 of predictors will be available at model implementation, and therefore we are restricted to using these when developing the model. Suppose this also reduces the expected C-statistic to 0.6. If we repeat the sample size calculations using $p=5$ and $C=0.6$, we find that ${N}_{original}=3236$ and ${N}_{sim}=2280$. The minimum required sample size from the simulation-based approach is now considerably smaller (30%) than the approach targeting the heuristic shrinkage factor. This is driven by the smaller C-statistic, and agrees with our simulation study results which suggest a smaller sample size than ${N}_{original}$ is needed in that situation.

## Discussion

### *Simulation study 1 (Compare performance of *${\widehat{S}}_{VH}$* and *${\widehat{S}}_{boot}$* as estimators of *${S}_{opt}$*)*

Simulation study 1 highlights the importance of using ${\widehat{S}}_{boot}$ as an estimator for the shrinkage factor as opposed to ${\widehat{S}}_{VH}$ when possible. While this is often recommended in guidance, the heuristic shrinkage factor ${\widehat{S}}_{VH}$ is still often applied during model development in applied articles [10–16], for reasons outlined in the introduction. It is already known that ${\widehat{S}}_{VH}$ is negatively correlated with the required amount of shrinkage, ${S}_{opt}$, but these simulation findings additionally show it is also a poor estimator of ${S}_{opt}$ on average (i.e. systematically biased, though the direction of bias depends on the scenario itself). Hence, for use after a model has been developed, it is unclear what positive attributes ${\widehat{S}}_{VH}$ has as an estimator of ${S}_{opt}$. While ${\widehat{S}}_{boot}$was biased in some scenarios, these were scenarios with very small N (< 150), and a high numbers of predictors (> 20) relative to the sample size (numbers of events). We emphasize that in such a setting, model developers should not be relying on applying shrinkage adjustments, as these have been shown to be inadequate to obtain desired model performance [3, 5]. Our results therefore further emphasise the importance of avoiding using the heuristic shrinkage factor after model development, and to use the bootstrap-derived global shrinkage factor instead if possible.

It should also be noted that the bias of the estimated shrinkage can be substantial; however, when the required shrinkage is large this bias may have limited influence on predictions because the regression coefficients are already heavily shrunk toward zero. In contrast, in scenarios where less shrinkage is appropriate, the bias may have a more pronounced impact.

### Simulation study 2 (compare performance of sample size criteria)

This simulation study acts as a replication/validation of the work of Pavlou et al., [19] using a broader data generating mechanism than the original study, and implemented by a distinct research group. This a key step when building an evidence base for new methods, and can be considered a phase II study from the “*phases of methodological research*” [25].

Simulation study 2 found that the mean amount of shrinkage required in models developed with sample size ${N}_{sim}$ was closer to 0.9 than those developed on sample size ${N}_{original}$. We therefore recommend the use of ${N}_{sim}$ when estimating sample size criteria for a clinical prediction model, although care may be needed if the expected C-statistic is very small ($< 0.6$).

Simulation study 2 used a similar underlying data generating mechanism to the study in which ${N}_{sim}$ was derived, but with a broader range of input parameters, allowing for up to 30 measured and 30 unmeasured predictor variables, and a wider range of outcome prevalences, and in data with predictors that had covariance. Simulation study 2 did not consider non-normal continuous predictor variables, binary predictor variables, or inaccurate estimates of the C-statistic when deriving the sample size, which are settings that should be considered important for future work.

Pavlou et al., [19] found that ${N}_{original}$ achieved the pre-specified shrinkage of 0.9 when the anticipated C-statistic was $<0.8$, however our simulation found this threshold to be closer to 0.73. This is evident from Fig. 8, which re-presents the data from Fig. 1, highlighting scenarios where the C-statistic is above or below 0.73. This is also evident from Table 3, where ${N}_{original}$ did not achieve a required shrinkage of 0.9 for the majority of models with $0.75\le {C}_{pop}<0.8$ (97.5th percentile of $mean({S}_{opt})=0.893$). Simulation study 2 also highlighted that for C-statistics below the threshold of 0.73, $mean\left({S}_{opt}\right)$ often exceeded 0.9 “too much” when using ${N}_{original}$. Such thresholds are likely be to closely tied to the data generating mechanism used to simulate the data and should be interpreted with caution.

## Concluding remarks

The aim of this study was to evaluate the bias of ${\widehat{S}}_{VH}$ as a predictor for ${S}_{opt}$. A natural follow-up from this was to evaluate the impact this had on the minimum sample size criteria of Riley et al. [17] and compare this with the recently suggested simulation based approach of Pavlou et al. [19] However, minimum sample size can also be estimated using an approach focused on the mean absolute prediction error [31]. The performance of this approach should also be compared with the approach of Pavlou et al. [19] in future work. This work should also act as a reminder not to develop complex clinical predictions models in small datasets. Both ${\widehat{S}}_{VH}$ and ${\widehat{S}}_{boot}$ performed poorly as estimators of ${S}_{opt}$ when there was a large amount of shrinkage required. Sufficient sample sizes are the only way to protect against this [17, 19, 32, 33], including limiting the number of predictor parameters relative to the number of anticipated outcome events.

The data underpinning the plots and tables presented for both simulations is available on GitHub [34]. Given the data could be presented in a number of ways, we encourage readers to download the simulation results themselves and test our conclusions. We found that when the when the C-statistic was large, ${\widehat{S}}_{VH}$ overestimated ${S}_{opt}$ on average, and when the C-statistic was small, ${\widehat{S}}_{VH}$ underestimated ${S}_{opt}$ on average. We found the threshold for under/overestimation to be 0.73, although this finding should be tested in more simulations with different data generating mechanisms. The further the C-statistic was from 0.73, the magnitude of the bias increased. The magnitude of the bias reduced the closer ${S}_{opt}$ was to 1, which is primarily achieved by increasing the sample size. We strongly recommend using a bootstrapped estimate of the global shrinkage factor (${\widehat{S}}_{boot}$) as opposed to the heuristic shrinkage factor (${\widehat{S}}_{VH}$) when possible, echoing existing recommendations [2, 8, 9]. An implication of this finding is that in scenarios where ${\widehat{S}}_{VH}$ is a poor estimator of ${S}_{opt}$, the sample size criteria of Riley et al., [17] may not result in the desired level of required shrinkage. Pavlou et al. [19] suggested an adjustment to the sample size criteria which we found to perform better. This should be used in conjunction with criteria 2 and 3 from the work of Riley et al. [17].

## Supplementary Information

Supplementary Material 1. Figures.

Supplementary Material 2. Figures under alternative DGM.

Supplementary Material 3. Tables and methods.

## Data Availability

The simulation was implemented in R version 4.3.1, [33] and rstudio [35] using the following packages: dplyr [36], ggplot2 [37], knitr [38], boot [39], rms [40], DescTools [41], bindata [42], pmsampsize [29], samplesizedev [43]. Code and simulation results are available from Manchester Predictive-Healthcare-Group public GitHub repository [34].

## References

[1] van Smeden M, Reitsma JB, Riley RD, et al. Clinical prediction models: diagnosis versus prognosis. J Clin Epidemiol. 2021;132:142–5. 33775387 10.1016/j.jclinepi.2021.01.009

[2] Harrell FE. Regression Modeling Strategies: With Applications to Linear Models, Logistic and Ordinal Regression, and Survival Analysis (2nd ed.). Springer Series in Statistics. Cham: Springer. ISBN: 978-3-319-19424-0 (Hardcover), 978-3-319-19425-7 (eBook). 2015.

[3] Riley RD, Snell KIE, Martin GP, et al. Penalization and shrinkage methods produced unreliable clinical prediction models especially when sample size was small. J Clin Epidemiol. 2021;132:88–96. 33307188 10.1016/j.jclinepi.2020.12.005 PMC8026952

[4] van Houwelingen JC. Shrinkage and penalized likelihood as methods to improve predictive accuracy. Stat Neerl. 2001;55:17–34.

[5] Calster BV, Smeden MV, Cock BD, et al. Regression shrinkage methods for clinical prediction models do not guarantee improved performance: simulation study. Stat Methods Med Res. 2020;29:3166–78. 32401702 10.1177/0962280220921415

[6] Martin GP, Riley RD, Collins GS, et al. Developing clinical prediction models when adhering to minimum sample size recommendations: the importance of quantifying bootstrap variability in tuning parameters and predictive performance. Stat Methods Med Res. 2021;30:2545–61. 34623193 10.1177/09622802211046388 PMC8649413

[7] Van Houwelingen J, Le Cessie S. Predictive value of statistical models. Stat Med. 1990;9:1303–25. 2277880 10.1002/sim.4780091109

[8] Steyerberg EW. Clinical prediction models: a practical approach to development, validation, and updating. 2nd ed. Springer Nature; 2019. 10.1007/978-3-030-16399-0.

[9] Riley RD, van der Windt D, Croft P, et al. Prognosis Research in Healthcare: Concepts, Methods, and Impact. Oxford: Oxford University Press; 2019.

[10] Kamtchum-Tatuene J, Saba L, Heldner MR, et al. Interleukin-6 predicts carotid plaque severity, vulnerability, and progression. Circ Res. 2022;131:E22–33. 35713008 10.1161/CIRCRESAHA.122.320877 PMC9308732

[11] Bonnett LJ, Kim L, Johnson A, et al. Risk of seizure recurrence in people with single seizures and early epilepsy – model development and external validation. Seizure. 2022;94:26–32. 34852983 10.1016/j.seizure.2021.11.007 PMC8776562

[12] Härmälä S, O’Brien A, Parisinos CA, et al. Development and validation of a prediction model to estimate the risk of liver cirrhosis in primary care patients with abnormal liver blood test results: protocol for an electronic health record study in Clinical Practice Research Datalink. Diagnostic Progn Res. 2019;3:1–11. 10.1186/s41512-019-0056-7 PMC6532213 31143841

[13] Siriussawakul A, Maboonyanon P, Kueprakone S, et al. Predictive performance of a multivariable difficult intubation model for obese patients. PLoS ONE. 2018;13:1–15. 10.1371/journal.pone.0203142 PMC6117055 30161197

[14] Ziauddeen N, Wilding S, Roderick PJ, et al. Predicting the risk of childhood overweight and obesity at 4–5 years using population-level pregnancy and early-life healthcare data. BMC Med. 2020;18:1–15. 32389121 10.1186/s12916-020-01568-z PMC7212594

[15] Fisher S, Manuel DG, Hsu AT, et al. Development and validation of a predictive algorithm for risk of dementia in the community setting. J Epidemiol Community Health. 2021;75:843–53. 34172513 10.1136/jech-2020-214797 PMC8372383

[16] Wahab RJ, Jaddoe VWV, van Klaveren D, et al. Preconception and early-pregnancy risk prediction for birth complications: development of prediction models within a population-based prospective cohort. BMC Pregnancy Childbirth. 2022;22:1–15. 35227240 10.1186/s12884-022-04497-2 PMC8886786

[17] Riley RD, Snell KIE, Ensor J, et al. Minimum sample size for developing a multivariable prediction model: part II - binary and time-to-event outcomes. Stat Med. 2019;38:1276–96. 30357870 10.1002/sim.7992 PMC6519266

[18] Riley RD, et al. Importance of sample size on the quality and utility of AI-based predictionmodels for healthcare. Lancet Digit. Health. 2025;7:100857. 10.1016/j.landig.2025.01.013 40461350

[19] Pavlou M, Ambler G, Qu C, et al. An evaluation of sample size requirements for developing risk prediction models with binary outcomes. BMC Med Res Methodol. 2024;24:1–13. 38987715 10.1186/s12874-024-02268-5 PMC11234534

[20] Efron B. Bootstrap methods: another look at the jackknife. Ann Stat. 1991;7:1–26.

[21] Morris TP, White IR, Crowther MJ. Using simulation studies to evaluate statistical methods. Stat Med. 2019;38:2074–102. 30652356 10.1002/sim.8086 PMC6492164

[22] Cox DR, Snell EJ. Analysis of Binary Data (2nd ed.). Chapman & Hall/CRC. ISBN 978-0412306204. 1989.

[23] Magee L. R2 measures based on wald and likelihood ratio joint significance tests. Am Stat. 1990;44:250–3.

[24] Hendry D, Nielsen B. Econoetric modeling: a likelihood approach. NJ: Princeton University Press; 2012.

[25] Heinze G, Boulesteix A-L, Kammer M, et al. Phases of methodological research in biostatistics - building the evidence base for new methods. Biometrical J. 2024;66:e2200222. 10.1002/bimj.202200222 PMC7615508 36737675

[26] Austin PC. The iterative bisection procedure: a useful tool for determining parameter values in data-generating processes in Monte Carlo simulations. BMC Med Res Methodol. 2023;23:1–10. 36800931 10.1186/s12874-023-01836-5 PMC9936690

[27] Johnson AEW, Pollard TJ, Shen L, et al. MIMIC-III, a freely accessible critical care database. Sci Data. 2016;3:1–9. 10.1038/sdata.2016.35 PMC4878278 27219127

[28] Riley RD, Ensor J, Snell KIE, et al. Calculating the sample size required for developing a clinical prediction model. BMJ. 2020;368:1–12. 10.1136/bmj.m441 32188600

[29] North RA, McCowan LME, Dekker GA, et al. Clinical risk prediction for pre-eclampsia in nulliparous women: Development of model in international prospective cohort. BMJ; 342. Epub ahead of print 2011. 10.1136/bmj.d1875. 10.1136/bmj.d1875 PMC3072235 21474517

[30] Ensor J, Martin EC, Riley RD. pmsampsize: Calculates the Minimum Sample Size Required for Developing a Multivariable Prediction Model. R package version 1.0.3. 2020. https://cran.r-project.org/package=pmsampsize.

[31] Van SM, Moons KGM, De GJAH, et al. Sample size for binary logistic prediction models: Beyond events per variable criteria. Stat Methods Med Res. 2019;28:2455–74. 29966490 10.1177/0962280218784726 PMC6710621

[32] Riley RD, Ensor J, Snell KIE. Calculating the sample size required for developing a clinical prediction model. BMJ. 2020;368:m441. 32188600 10.1136/bmj.m441

[33] Riley RD, Whittle R, Sadatsafavi M, Martin GP, Pate A, Collins GS, Ensor J. A general sample size framework for developing or updating a clinical prediction model. arXiv. 2025. 10.48550/arXiv.2504.18730. 10.1186/s12874-026-02856-7 PMC13196178 42056904

[34] Pate A. GitHub repository. Manchester Predictive Healthcare Group. MRC-Multi-Outcome-Project-5-heuristic-shrinkage-factor. 2025. https://github.com/manchester-predictive-healthcare-group/CHI-MRC-multi-outcome/tree/main/Project%205%20heuristic%20shrinkage%20factor 5 heuristic shrinkage factor. Accessed 21 Feb 2025.

[35] RStudio: Integrated Development for R. RStudio Team. 2020. http://www.rstudio.com/.

[36] Wickham H, Francois R, Henry L, et al. dplyr: A Grammar of Data Manipulation.

[37] Wickham H. ggplot2: Elegant Graphics for Data Analysis. 2016. https://ggplot2.tidyverse.org.

[38] Xie Y. knitr: A General-Purpose Package for Dynamic Report Generation in R. 2021. https://cran.r-project.org/package=knitr.

[39] Canty A, Ripley B. boot: Bootstrap R (S-Plus) Functions. 2022. https://cran.r-project.org/package=boot.

[40] Harrell Jr FE. rms: Regression Modeling Strategies.

[41] Al.} S {Andri et mult. {DescTools}: Tools for Descriptive Statistics. 2021. https://cran.r-project.org/package=DescTools.

[42] Leisch F, Weingessel A, Hornik K. _bindata: Generation of Artificial Binary Data_. 2024. https://cran.r-project.org/package=bindata.

[43] Pavlou M. samplesizedev: sample size for development of risk models with binary and survival outcomes. 2024. https://github.com/mpavlou/samplesizedev.

