# Correction: Agreement between heuristic shrinkage factor and optimal shrinkage factors in logistic regression for risk prediction: a simulation study across different sample sizes and settings

**DOI:** 10.1186/s41512-026-00232-z

**Published:** 2026-07-07

**Authors:** Alexander Pate, Glen P. Martin, Richard D. Riley

**Affiliations:** 1 Centre for Informatics, Imaging and Data Science, Faculty of Biology, Medicine and Health, University of Manchester, Manchester, UK; 2 Department of Applied Health Sciences, School of Health Sciences, College of Medicine and Health, University of Birmingham, Birmingham, B15 2TT UK; 3 National Institute for Health and Care Research (NIHR) Birmingham Biomedical Research Centre, Birmingham, UK

**Correction: Diagn Progn Res 10, 15 (2026)**

**https://doi.org/10.1186/s41512-026-00222-1**

Following publication of the original article [1], the author reported a typesetting error, whereby the abstract was erroneously omitted.

The article’s abstract is as follows:

**Abstract**

**Introduction**: The heuristic shrinkage factor of Van Houwelingen and Le Cessie ($\:{S}_{VH}$) is a commonly used closed-form solution to adjust for overfitting in unpenalised logistic regression models for risk prediction. It is also the basis of widely-adopted minimum sample size criteria for developing clinical prediction models. However, current evidence is lacking regarding the bias of $\:{S}_{VH}$ compared to the optimal shrinkage factor ($\:{S}_{opt})$. Here, we examine this issue and also assess the bias of an alternative bootstrap-derived shrinkage factor ($\:{S}_{boot}$).

**Methods**: We undertook two simulation studies. The first examined the bias of $\:{S}_{VH}$ and $\:{S}_{boot}$ as estimators of $\:{S}_{opt}$ across a range of different scenarios defined by $\:{C}_{pop}$, the C-statistic of the model developed in a population sized dataset. The second examined the bias of $\:{S}_{opt}$ when using development sample sizes targeting a shrinkage of 0.9, based on a sample size calculation defined by $\:{S}_{VH}$ itself ($\:{N}_{original}$) or by an adapted simulation-based approach ($\:{N}_{sim}$).

**Results**: For high C-statistics, $\:{S}_{VH}$ overestimated $\:{S}_{opt}$, whereas for low C-statistics $\:{S}_{VH}$ underestimates $\:{S}_{opt}$. For example, across scenarios when $\:0.8\le\:{C}_{pop}<0.85$, the 95-percentile range in the bias was ($\:0.005,\:0.387$), compared to $\:(-0.580,\:-0.007)$ across scenarios when $\:0.6\le\:{C}_{pop}<0.65$. The magnitude of bias increased as $\:{C}_{pop}$ tended to either $\:0.5$ or $\:1$. As sample size increased and $\:{S}_{opt}\to\:1$, the magnitude of the bias in either direction reduced. $\:{S}_{boot}$ was less biased than $\:{S}_{VH}$, with a median magnitude of bias across all scenarios of $\:0.007$, compared to $\:0.032$ for $\:{S}_{VH}$. Developing models on datasets of size $\:{N}_{sim}$ gave $\:mean\left({S}_{opt}\right)$ closer to 0.9 (mean magnitude of bias across all scenarios $\:0.004$) than $\:{N}_{original}$ (mean magnitude of bias $\:0.041$).

**Conclusions**: $\:{S}_{VH}$ is often a poor estimator of the optimal global shrinkage factor. If global shrinkage is needed, we recommend using the bootstrap shrinkage estimate. The bootstrap estimate shows minimal bias in most scenarios, though in small samples the variability is large so provides no guarantees to address overfitting in a single dataset. A sample size calculation based on simulation is often preferable over formula dependent on targeting $\:{S}_{VH}$.

The original article [1] has been updated.
