# Supplementary material for: Agreement between heuristic shrinkage factor and optimal shrinkage factors in logistic regression for risk prediction: a simulation study across different sample sizes and settings
Source: Diagn Progn Res. 2026 May 18;10:15. doi: 10.1186/s41512-026-00222-1 (PMC13182129; doi:10.1186/s41512-026-00222-1)
Supplement: Supplementary file 1 — Supplementary Material 1. Figures. [file 41512_2026_222_MOESM1_ESM.html]

Supplementary Figures


# Supplementary Figures

#### Alex Pate

#### 2024-09-27

Associated with manuscript: Agreement between heuristic shrinkage
factor and optimal shrinkage factors in logistic regression for risk
prediction: a simulation study across different sample sizes and
settings

# 1 Plots for results from simulation study 1

We present plots of \(\hat{S}\_{VH}\)
and \(\hat{S}\_{boot}\) plotted against
\(S\_{opt}\) from simulation study 1 for
every combination of the simulation inputs:

- Scenarios where data was simulated with a zero-covariance
  matrix
- Plots presented by \(R^{2}\_{CS,app}\) and \(C\_{app}\), as opposed to \(R^{2}\_{CS,pop}\) and \(C\_{pop}\).
- Median instead of mean

**Figure S1: mean(\(\hat{S}\_{VH}\)) plotted against mean(\(S\_{opt}\)), non-zero covariance in DGM,
presented with respect to population-level model
performance**

*Estimator = mean(\(\hat{S}\_{VH}\))*

*Covariance structure = non-zero covariance in DGM*

*By variable = presented with respect to population-level model
performance*

---

**Figure S2: mean(\(\hat{S}\_{VH}\)) plotted against mean(\(S\_{opt}\)), non-zero covariance in DGM,
presented with respect to apparent model performance**

*Estimator = mean(\(\hat{S}\_{VH}\))*

*Covariance structure = non-zero covariance in DGM*

*By variable = presented with respect to apparent model
performance*

---

**Figure S3: median(\(\hat{S}\_{VH}\)) plotted against
median(\(S\_{opt}\)), non-zero
covariance in DGM, presented with respect to population-level model
performance**

*Estimator = median(\(\hat{S}\_{VH}\))*

*Covariance structure = non-zero covariance in DGM*

*By variable = presented with respect to population-level model
performance*

---

**Figure S4: median(\(\hat{S}\_{VH}\)) plotted against
median(\(S\_{opt}\)), non-zero
covariance in DGM, presented with respect to apparent model
performance**

*Estimator = median(\(\hat{S}\_{VH}\))*

*Covariance structure = non-zero covariance in DGM*

*By variable = presented with respect to apparent model
performance*

---

**Figure S5: mean(\(\hat{S}\_{boot}\)) plotted against
mean(\(S\_{opt}\)), non-zero covariance
in DGM, presented with respect to population-level model
performance**

*Estimator = mean(\(\hat{S}\_{boot}\))*

*Covariance structure = non-zero covariance in DGM*

*By variable = presented with respect to population-level model
performance*

---

**Figure S6: mean(\(\hat{S}\_{boot}\)) plotted against
mean(\(S\_{opt}\)), non-zero covariance
in DGM, presented with respect to apparent model
performance**

*Estimator = mean(\(\hat{S}\_{boot}\))*

*Covariance structure = non-zero covariance in DGM*

*By variable = presented with respect to apparent model
performance*

---

**Figure S7: median(\(\hat{S}\_{boot}\)) plotted against
median(\(S\_{opt}\)), non-zero
covariance in DGM, presented with respect to population-level model
performance**

*Estimator = median(\(\hat{S}\_{boot}\))*

*Covariance structure = non-zero covariance in DGM*

*By variable = presented with respect to population-level model
performance*

---

**Figure S8: median(\(\hat{S}\_{boot}\)) plotted against
median(\(S\_{opt}\)), non-zero
covariance in DGM, presented with respect to apparent model
performance**

*Estimator = median(\(\hat{S}\_{boot}\))*

*Covariance structure = non-zero covariance in DGM*

*By variable = presented with respect to apparent model
performance*

---

**Figure S9: mean(\(\hat{S}\_{VH}\)) plotted against mean(\(S\_{opt}\)), zero covariance in DGM,
presented with respect to population-level model
performance**

*Estimator = mean(\(\hat{S}\_{VH}\))*

*Covariance structure = zero covariance in DGM*

*By variable = presented with respect to population-level model
performance*

---

**Figure S10: mean(\(\hat{S}\_{VH}\)) plotted against mean(\(S\_{opt}\)), zero covariance in DGM,
presented with respect to apparent model performance**

*Estimator = mean(\(\hat{S}\_{VH}\))*

*Covariance structure = zero covariance in DGM*

*By variable = presented with respect to apparent model
performance*

---

**Figure S11: median(\(\hat{S}\_{VH}\)) plotted against
median(\(S\_{opt}\)), zero covariance in
DGM, presented with respect to population-level model
performance**

*Estimator = median(\(\hat{S}\_{VH}\))*

*Covariance structure = zero covariance in DGM*

*By variable = presented with respect to population-level model
performance*

---

**Figure S12: median(\(\hat{S}\_{VH}\)) plotted against
median(\(S\_{opt}\)), zero covariance in
DGM, presented with respect to apparent model performance**

*Estimator = median(\(\hat{S}\_{VH}\))*

*Covariance structure = zero covariance in DGM*

*By variable = presented with respect to apparent model
performance*

---

**Figure S13: mean(\(\hat{S}\_{boot}\)) plotted against
mean(\(S\_{opt}\)), zero covariance in
DGM, presented with respect to population-level model
performance**

*Estimator = mean(\(\hat{S}\_{boot}\))*

*Covariance structure = zero covariance in DGM*

*By variable = presented with respect to population-level model
performance*

---

**Figure S14: mean(\(\hat{S}\_{boot}\)) plotted against
mean(\(S\_{opt}\)), zero covariance in
DGM, presented with respect to apparent model performance**

*Estimator = mean(\(\hat{S}\_{boot}\))*

*Covariance structure = zero covariance in DGM*

*By variable = presented with respect to apparent model
performance*

---

**Figure S15: median(\(\hat{S}\_{boot}\)) plotted against
median(\(S\_{opt}\)), zero covariance in
DGM, presented with respect to population-level model
performance**

*Estimator = median(\(\hat{S}\_{boot}\))*

*Covariance structure = zero covariance in DGM*

*By variable = presented with respect to population-level model
performance*

---

**Figure S16: median(\(\hat{S}\_{boot}\)) plotted against
median(\(S\_{opt}\)), zero covariance in
DGM, presented with respect to apparent model performance**

*Estimator = median(\(\hat{S}\_{boot}\))*

*Covariance structure = zero covariance in DGM*

*By variable = presented with respect to apparent model
performance*

---

**Figure S17: mean(\(\hat{S}\_{VH}\)) plotted against mean(\(S\_{opt}\)), grouped by mean(\(C\_{app}\)), non-zero covariance in
DGM**

**Figure S18: mean(\(\hat{S}\_{VH}\)) plotted against mean(\(S\_{opt}\)), grouped by \(C\_{pop}\), non-zero covariance in
DGM**

**Figure S19: mean(\(\hat{S}\_{VH}\)) plotted against mean(\(S\_{opt}\)), grouped by mean(\(C\_{app}\)), zero covariance in
DGM**

**Figure S20: mean(\(\hat{S}\_{VH}\)) plotted against mean(\(S\_{opt}\)), grouped by \(C\_{pop}\), zero covariance in
DGM**

# 2 Plots for standard deviation results from simulation study 1

We present plots of the standard deviation of \(\hat{S}\_{VH}\) and \(\hat{S}\_{boot}\) plotted against the
standard deviation of \(S\_{opt}\) from
simulation study 1. We remind readers we do not expect to see a standard
deviation of zero, given that \(S\_{opt}\) itself has variation across
simulation iterations.

**Figure S21: sd(\(\hat{S}\_{VH}\)) plotted against sd(\(S\_{opt}\)), non-zero covariance in DGM,
presented with respect to population-level model
performance**

*Estimator = sd(\(\hat{S}\_{VH}\))*

*Covariance structure = non-zero covariance in DGM*

*By variable = presented with respect to population-level model
performance*

---

**Figure S22: sd(\(\hat{S}\_{boot}\)) plotted against sd(\(S\_{opt}\)), non-zero covariance in DGM,
presented with respect to population-level model
performance**

*Estimator = sd(\(\hat{S}\_{boot}\))*

*Covariance structure = non-zero covariance in DGM*

*By variable = presented with respect to population-level model
performance*

---

**Figure S23: sd(\(\hat{S}\_{VH}\)) plotted against sd(\(S\_{opt}\)), zero covariance in DGM,
presented with respect to population-level model
performance**

*Estimator = sd(\(\hat{S}\_{VH}\))*

*Covariance structure = zero covariance in DGM*

*By variable = presented with respect to population-level model
performance*

---

**Figure S24: sd(\(\hat{S}\_{boot}\)) plotted against sd(\(S\_{opt}\)), zero covariance in DGM,
presented with respect to population-level model
performance**

*Estimator = sd(\(\hat{S}\_{boot}\))*

*Covariance structure = zero covariance in DGM*

*By variable = presented with respect to population-level model
performance*

---

# 3 Simulation study 2

We present a plot of \(N\_{sim}\)
against \(N\_{original}\):

**Figure S25: \(N\_{sim}\)
plotted against \(N\_{original}\)**

We also present plots for the instabilty of \(S\_{opt}\) in a range of scenarios meeting
the sample size criteria for \(N\_{original}\) with different sample
sizes:

**Figure S26: Equivalent of Figure 5 from nanuscript but when
criteria is met for \(N\_{original}\)**
