# Supplementary material for: Agreement between heuristic shrinkage factor and optimal shrinkage factors in logistic regression for risk prediction: a simulation study across different sample sizes and settings
Source: Diagn Progn Res. 2026 May 18;10:15. doi: 10.1186/s41512-026-00222-1 (PMC13182129; doi:10.1186/s41512-026-00222-1)
Supplement: Supplementary file 2 — Supplementary Material 2. Figures under alternative DGM. [file 41512_2026_222_MOESM2_ESM.html]

Supplementary Figures (alternative data generating mechanism)


# Supplementary Figures (alternative data generating mechanism)

#### Alex Pate

#### 2024-09-27

Associated with manuscript: Agreement between heuristic shrinkage
factor and optimal shrinkage factors in logistic regression for risk
prediction: a simulation study across different sample sizes and
settings

This supplementary data file contains results for the alternative
data generating mechanism, where \(Q\_{meas}\) and the C-statistic are
independent - see section “Impact of using a different data generating
mechanism” from the main manuscript).

# 1 Plots for results from simulation study 1

We present plots of \(\hat{S}\_{VH}\)
and \(\hat{S}\_{boot}\) plotted against
\(S\_{opt}\) from simulation study 1 for
every combination of the simulation inputs, where the alternative data
generating mechanism was used (see section 4 for more details).

**Figure S27: alternative DGM (see section 4 of manuscript);
mean(\(\hat{S}\_{VH}\)) plotted against
mean(\(S\_{opt}\)), non-zero covariance
in DGM, presented with respect to population-level model
performance**

*Estimator = mean(\(\hat{S}\_{VH}\))*

*Covariance structure = non-zero covariance in DGM*

*By variable = presented with respect to population-level model
performance*

---

**Figure S28: alternative DGM (see section 4 of manuscript);
mean(\(\hat{S}\_{VH}\)) plotted against
mean(\(S\_{opt}\)), non-zero covariance
in DGM, presented with respect to apparent model
performance**

*Estimator = mean(\(\hat{S}\_{VH}\))*

*Covariance structure = non-zero covariance in DGM*

*By variable = presented with respect to apparent model
performance*

---

**Figure S29: alternative DGM (see section 4 of manuscript);
median(\(\hat{S}\_{VH}\)) plotted
against median(\(S\_{opt}\)), non-zero
covariance in DGM, presented with respect to population-level model
performance**

*Estimator = median(\(\hat{S}\_{VH}\))*

*Covariance structure = non-zero covariance in DGM*

*By variable = presented with respect to population-level model
performance*

---

**Figure S30: alternative DGM (see section 4 of manuscript);
median(\(\hat{S}\_{VH}\)) plotted
against median(\(S\_{opt}\)), non-zero
covariance in DGM, presented with respect to apparent model
performance**

*Estimator = median(\(\hat{S}\_{VH}\))*

*Covariance structure = non-zero covariance in DGM*

*By variable = presented with respect to apparent model
performance*

---

**Figure S31: alternative DGM (see section 4 of manuscript);
mean(\(\hat{S}\_{boot}\)) plotted
against mean(\(S\_{opt}\)), non-zero
covariance in DGM, presented with respect to population-level model
performance**

*Estimator = mean(\(\hat{S}\_{boot}\))*

*Covariance structure = non-zero covariance in DGM*

*By variable = presented with respect to population-level model
performance*

---

**Figure S32: alternative DGM (see section 4 of manuscript);
mean(\(\hat{S}\_{boot}\)) plotted
against mean(\(S\_{opt}\)), non-zero
covariance in DGM, presented with respect to apparent model
performance**

*Estimator = mean(\(\hat{S}\_{boot}\))*

*Covariance structure = non-zero covariance in DGM*

*By variable = presented with respect to apparent model
performance*

---

**Figure S33: alternative DGM (see section 4 of manuscript);
median(\(\hat{S}\_{boot}\)) plotted
against median(\(S\_{opt}\)), non-zero
covariance in DGM, presented with respect to population-level model
performance**

*Estimator = median(\(\hat{S}\_{boot}\))*

*Covariance structure = non-zero covariance in DGM*

*By variable = presented with respect to population-level model
performance*

---

**Figure S34: alternative DGM (see section 4 of manuscript);
median(\(\hat{S}\_{boot}\)) plotted
against median(\(S\_{opt}\)), non-zero
covariance in DGM, presented with respect to apparent model
performance**

*Estimator = median(\(\hat{S}\_{boot}\))*

*Covariance structure = non-zero covariance in DGM*

*By variable = presented with respect to apparent model
performance*

---

**Figure S35: alternative DGM (see section 4 of manuscript);
mean(\(\hat{S}\_{VH}\)) plotted against
mean(\(S\_{opt}\)), zero covariance in
DGM, presented with respect to population-level model
performance**

*Estimator = mean(\(\hat{S}\_{VH}\))*

*Covariance structure = zero covariance in DGM*

*By variable = presented with respect to population-level model
performance*

---

**Figure S36: alternative DGM (see section 4 of manuscript);
mean(\(\hat{S}\_{VH}\)) plotted against
mean(\(S\_{opt}\)), zero covariance in
DGM, presented with respect to apparent model performance**

*Estimator = mean(\(\hat{S}\_{VH}\))*

*Covariance structure = zero covariance in DGM*

*By variable = presented with respect to apparent model
performance*

---

**Figure S37: alternative DGM (see section 4 of manuscript);
median(\(\hat{S}\_{VH}\)) plotted
against median(\(S\_{opt}\)), zero
covariance in DGM, presented with respect to population-level model
performance**

*Estimator = median(\(\hat{S}\_{VH}\))*

*Covariance structure = zero covariance in DGM*

*By variable = presented with respect to population-level model
performance*

---

**Figure S38: alternative DGM (see section 4 of manuscript);
median(\(\hat{S}\_{VH}\)) plotted
against median(\(S\_{opt}\)), zero
covariance in DGM, presented with respect to apparent model
performance**

*Estimator = median(\(\hat{S}\_{VH}\))*

*Covariance structure = zero covariance in DGM*

*By variable = presented with respect to apparent model
performance*

---

**Figure S39: alternative DGM (see section 4 of manuscript);
mean(\(\hat{S}\_{boot}\)) plotted
against mean(\(S\_{opt}\)), zero
covariance in DGM, presented with respect to population-level model
performance**

*Estimator = mean(\(\hat{S}\_{boot}\))*

*Covariance structure = zero covariance in DGM*

*By variable = presented with respect to population-level model
performance*

---

**Figure S40: alternative DGM (see section 4 of manuscript);
mean(\(\hat{S}\_{boot}\)) plotted
against mean(\(S\_{opt}\)), zero
covariance in DGM, presented with respect to apparent model
performance**

*Estimator = mean(\(\hat{S}\_{boot}\))*

*Covariance structure = zero covariance in DGM*

*By variable = presented with respect to apparent model
performance*

---

**Figure S41: alternative DGM (see section 4 of manuscript);
median(\(\hat{S}\_{boot}\)) plotted
against median(\(S\_{opt}\)), zero
covariance in DGM, presented with respect to population-level model
performance**

*Estimator = median(\(\hat{S}\_{boot}\))*

*Covariance structure = zero covariance in DGM*

*By variable = presented with respect to population-level model
performance*

---

**Figure S42: alternative DGM (see section 4 of manuscript);
median(\(\hat{S}\_{boot}\)) plotted
against median(\(S\_{opt}\)), zero
covariance in DGM, presented with respect to apparent model
performance**

*Estimator = median(\(\hat{S}\_{boot}\))*

*Covariance structure = zero covariance in DGM*

*By variable = presented with respect to apparent model
performance*

---

**Figure S43: mean(\(\hat{S}\_{VH}\)) plotted against mean(\(S\_{opt}\)), grouped by mean(\(C\_{app}\)), non-zero covariance in
DGM**

**Figure S44: mean(\(\hat{S}\_{VH}\)) plotted against mean(\(S\_{opt}\)), grouped by \(C\_{pop}\), non-zero covariance in
DGM**

**Figure S45: mean(\(\hat{S}\_VH\)) plotted against mean(\(S\_{opt}\)), grouped by mean(\(C\_{app}\)), zero covariance in
DGM**

**Figure S46: mean(\(\hat{S}\_{VH}\)) plotted against mean(\(S\_{opt}\)), grouped by \(C\_{pop}\), zero covariance in
DGM**

# 2 Simulation study 2

We present a plot of \(N\_{sim}\)
against \(N\_{original}\):

**Figure S47: \(N\_{sim}\)
plotted against \(N\_{original}\)**
