# Supplementary material for: Agreement between heuristic shrinkage factor and optimal shrinkage factors in logistic regression for risk prediction: a simulation study across different sample sizes and settings
Source: Diagn Progn Res. 2026 May 18;10:15. doi: 10.1186/s41512-026-00222-1 (PMC13182129; doi:10.1186/s41512-026-00222-1)
Supplement: Supplementary file 3 — Supplementary Material 3. Tables and methods. [file 41512_2026_222_MOESM3_ESM.docx]

Supplementary Tables for Manuscript: Agreement between heuristic shrinkage factor and optimal shrinkage factors in logistic regression for risk prediction: a simulation study across different sample sizes and settings

Alexander Pate, Glen P Martin, Richard R Riley

# Supplementary Tables – Simulation Study 1

*Supplementary Table 1: Simulation Study 1 - The mean, and 2.5^th^, 25^th^, 50^th^, 75^th^ and 97.5^th^ percentiles of the bias of* $S_{VH}$ *and* $S_{boot}$ *across scenarios where data was simulated with a non-zero covariance structure, stratified by* $N$

|  | **2.5%** | **25%** | **50%** | **75%** | **97.5%** | **Mean (sd)** |
| --- | --- | --- | --- | --- | --- | --- |
| $\boldsymbol{100\leq N<200}$ **(3,213 scenarios)** | | | | | | |
| **Bias of** $\boldsymbol{S}_{\boldsymbol{VH}}$ | -0.390 | -0.013 | 0.121 | 0.229 | 0.449 | 0.097 (0.205) |
| **Bias of** $\boldsymbol{S}_{\boldsymbol{boot}}$ | -0.186 | -0.077 | 0.007 | 0.043 | 0.125 | -0.016 (0.086) |
| $\boldsymbol{200\leq N<500}$ **(2,756 scenarios)** | | | | | | |
| **Bias of** $\boldsymbol{S}_{\boldsymbol{VH}}$ | -0.298 | -0.014 | 0.049 | 0.099 | 0.180 | 0.026 (0.126) |
| **Bias of** $\boldsymbol{S}_{\boldsymbol{boot}}$ | -0.035 | 0.003 | 0.014 | 0.026 | 0.072 | 0.015 (0.030) |
| $\boldsymbol{500\leq N<1,000}$ **(2,420 scenarios)** | | | | | | |
| **Bias of** $\boldsymbol{S}_{\boldsymbol{VH}}$ | -0.210 | -0.009 | 0.021 | 0.045 | 0.083 | 0.003 (0.090) |
| **Bias of** $\boldsymbol{S}_{\boldsymbol{boot}}$ | -0.015 | 0.000 | 0.005 | 0.011 | 0.032 | 0.006 (0.022) |
| $\boldsymbol{1,000\leq N<2,500}$ **(3,337 scenarios)** | | | | | | |
| **Bias of** $\boldsymbol{S}_{\boldsymbol{VH}}$ | -0.116 | -0.006 | 0.009 | 0.020 | 0.040 | -0.003 (0.064) |
| **Bias of** $\boldsymbol{S}_{\boldsymbol{boot}}$ | -0.012 | -0.002 | 0.001 | 0.005 | 0.016 | 0.001 (0.019) |
| $\boldsymbol{2,500\leq N<5,000}$ **(2,430 scenarios)** | | | | | | |
| **Bias of** $\boldsymbol{S}_{\boldsymbol{VH}}$ | -0.060 | -0.003 | 0.004 | 0.010 | 0.019 | -0.002 (0.037) |
| **Bias of** $\boldsymbol{S}_{\boldsymbol{boot}}$ | -0.010 | -0.002 | 0.000 | 0.003 | 0.011 | 0.000 (0.017) |

*Supplementary Table 2: Simulation Study 1 - The mean, and 2.5^th^, 25^th^, 50^th^, 75^th^ and 97.5^th^ percentiles of the bias of* $S_{VH}$ *and* $S_{boot}$ *across scenarios where data was simulated with a zero covariance structure, stratified by* $N$

|  | **2.5%** | **25%** | **50%** | **75%** | **97.5%** | **Mean (sd)** |
| --- | --- | --- | --- | --- | --- | --- |
| $\boldsymbol{100\leq N<200}$ **(3,114 scenarios)** | | | | | | |
| **Bias of** $\boldsymbol{S}_{\boldsymbol{VH}}$ | -0.377 | -0.032 | 0.089 | 0.185 | 0.364 | 0.061 (0.186) |
| **Bias of** $\boldsymbol{S}_{\boldsymbol{boot}}$ | -0.146 | -0.026 | 0.047 | 0.088 | 0.170 | 0.030 (0.085) |
| $\boldsymbol{200\leq}\boldsymbol{C}_{\boldsymbol{pop}}\boldsymbol{<500}$ **(2,629 scenarios)** | | | | | | |
| **Bias of** $\boldsymbol{S}_{\boldsymbol{VH}}$ | -0.383 | -0.042 | 0.027 | 0.074 | 0.151 | -0.008 (0.142) |
| **Bias of** $\boldsymbol{S}_{\boldsymbol{boot}}$ | -0.022 | 0.018 | 0.031 | 0.048 | 0.099 | 0.034 (0.035) |
| $\boldsymbol{500\leq N<1,000}$ **(2,286 scenarios)** | | | | | | |
| **Bias of** $\boldsymbol{S}_{\boldsymbol{VH}}$ | -0.274 | -0.024 | 0.011 | 0.035 | 0.068 | -0.015 (0.098) |
| **Bias of** $\boldsymbol{S}_{\boldsymbol{boot}}$ | -0.016 | 0.004 | 0.011 | 0.019 | 0.044 | 0.012 (0.036) |
| $\boldsymbol{1,000\leq N<2,500}$ **(3,333 scenarios)** | | | | | | |
| **Bias of** $\boldsymbol{S}_{\boldsymbol{VH}}$ | -0.136 | -0.015 | 0.004 | 0.016 | 0.035 | -0.011 (0.071) |
| **Bias of** $\boldsymbol{S}_{\boldsymbol{boot}}$ | -0.013 | -0.001 | 0.003 | 0.008 | 0.021 | 0.003 (0.020) |
| $\boldsymbol{2,500\leq N<5,000}$ **(2,539 scenarios)** | | | | | | |
| **Bias of** $\boldsymbol{S}_{\boldsymbol{VH}}$ | -0.080 | -0.007 | 0.002 | 0.008 | 0.016 | -0.007 (0.046) |
| **Bias of** $\boldsymbol{S}_{\boldsymbol{boot}}$ | -0.012 | -0.002 | 0.001 | 0.004 | 0.012 | 0.001 (0.015) |

*Supplementary Table 3 - Simulation Study 1: The mean, and 2.5^th^, 25^th^, 50^th^, 75^th^ and 97.5^th^ percentiles of the bias of* $S_{VH}$ *and* $S_{boot}$ *across scenarios where data was simulated with a zero covariance structure, stratified by* $C_{pop}$

|  | **2.5%** | **25%** | **50%** | **75%** | **97.5%** | **Mean (sd)** |
| --- | --- | --- | --- | --- | --- | --- |
| **All (13,901)** | | | | | | |
| **Magnitude of bias of** $\boldsymbol{S}_{\boldsymbol{VH}}$ | 0.001 | 0.010 | 0.029 | 0.088 | 0.355 | 0.071 (0.103) |
| **Magnitude of bias of** $\boldsymbol{S}_{\boldsymbol{boot}}$ | 0.000 | 0.004 | 0.012 | 0.040 | 0.140 | 0.030 (0.042) |
| **All (13,901)** | | | | | | |
| **Bias of** $\boldsymbol{S}_{\boldsymbol{VH}}$ | -0.303 | -0.017 | 0.008 | 0.041 | 0.251 | 0.006 (0.125) |
| **Bias of** $\boldsymbol{S}_{\boldsymbol{boot}}$ | -0.101 | -0.000 | 0.007 | 0.030 | 0.126 | 0.016 (0.049) |
| $\boldsymbol{0.6\leq}\boldsymbol{C}_{\boldsymbol{pop}}\boldsymbol{<0.65}$ **(1,460 scenarios)** | | | | | | |
| **Bias of** $\boldsymbol{S}_{\boldsymbol{VH}}$ | -0.500 | -0.185 | -0.076 | -0.032 | -0.007 | -0.131 (0.140) |
| **Bias of** $\boldsymbol{S}_{\boldsymbol{boot}}$ | -0.027 | -0.002 | 0.008 | 0.034 | 0.184 | 0.028 (0.061) |
| $\boldsymbol{0.65\leq}\boldsymbol{C}_{\boldsymbol{pop}}\boldsymbol{<0.7}$ **(2,594 scenarios)** | | | | | | |
| **Bias of** $\boldsymbol{S}_{\boldsymbol{VH}}$ | -0.244 | -0.053 | -0.019 | -0.006 | 0.029 | -0.041 (0.066) |
| **Bias of** $\boldsymbol{S}_{\boldsymbol{boot}}$ | -0.010 | 0.001 | 0.011 | 0.058 | 0.151 | 0.035 (0.048) |
| $\boldsymbol{0.7\leq}\boldsymbol{C}_{\boldsymbol{pop}}\boldsymbol{<0.75}$ **(4,117 scenarios)** | | | | | | |
| **Bias of** $\boldsymbol{S}_{\boldsymbol{VH}}$ | -0.042 | -0.001 | 0.009 | 0.033 | 0.170 | 0.024 (0.052) |
| **Bias of** $\boldsymbol{S}_{\boldsymbol{boot}}$ | -0.012 | 0.002 | 0.011 | 0.046 | 0.100 | 0.025 (0.033) |
| $\boldsymbol{0.75\leq}\boldsymbol{C}_{\boldsymbol{pop}}\boldsymbol{<0.8}$ **(4,323 scenarios)** | | | | | | |
| **Bias of** $\boldsymbol{S}_{\boldsymbol{VH}}$ | 0.001 | 0.014 | 0.036 | 0.106 | 0.289 | 0.072 (0.083) |
| **Bias of** $\boldsymbol{S}_{\boldsymbol{boot}}$ | -0.119 | -0.001 | 0.006 | 0.020 | 0.052 | 0.001 (0.038) |
| $\boldsymbol{0.8\leq}\boldsymbol{C}_{\boldsymbol{pop}}\boldsymbol{<0.85}$ **(884 scenarios)** | | | | | | |
| **Bias of** $\boldsymbol{S}_{\boldsymbol{VH}}$ | 0.007 | 0.022 | 0.051 | 0.164 | 0.412 | 0.109 (0.120) |
| **Bias of** $\boldsymbol{S}_{\boldsymbol{boot}}$ | -0.166 | -0.007 | 0.002 | 0.008 | 0.024 | -0.021 (0.054) |
| $\boldsymbol{0.85\leq}\boldsymbol{C}_{\boldsymbol{pop}}\boldsymbol{<0.9}$ **(4 scenarios)** | | | | | | |
| **Bias of** $\boldsymbol{S}_{\boldsymbol{VH}}$ | 0.045 | 0.084 | 0.123 | 0.194 | 0.316 | 0.154 (0.125) |
| **Bias of** $\boldsymbol{S}_{\boldsymbol{boot}}$ | -0.168 | -0.044 | 0.004 | 0.008 | 0.012 | -0.040 (0.094) |

Note there are very few scenarios where $0.85\leq C_{pop}<0.9$ for this data generating mechanism.

*Supplementary Table 4: Simulation Study 1: 50^th^, 90^th^ and 99^th^ percentiles of the Monte Carlo Standard Error (MCE) of the bias of* $S_{VH}$ *and* $S_{boot}$ *across scenarios where data was simulated with a zero covariance structure, stratified by* $C_{pop}$

|  | **50%** | **90%** | **99%** |
| --- | --- | --- | --- |
| **All (13,901 scenarios)** | | | |
| **MCE of bias of** $\boldsymbol{S}_{\boldsymbol{VH}}$ | 0.008 | 0.021 | 0.072 |
| **MCE of bias of** $\boldsymbol{S}_{\boldsymbol{boot}}$ | 0.006 | 0.013 | 0.033 |
| $\boldsymbol{0.6\leq}\boldsymbol{C}_{\boldsymbol{pop}}\boldsymbol{<0.65}$ **(1,460 scenarios)** | | | |
| **MCE of bias of** $\boldsymbol{S}_{\boldsymbol{VH}}$ | 0.014 | 0.050 | 0.106 |
| **MCE of bias of** $\boldsymbol{S}_{\boldsymbol{boot}}$ | 0.011 | 0.022 | 0.049 |
| $\boldsymbol{0.65\leq}\boldsymbol{C}_{\boldsymbol{pop}}\boldsymbol{<0.7}$ **(2,594 scenarios)** | | | |
| **MCE of bias of** $\boldsymbol{S}_{\boldsymbol{VH}}$ | 0.010 | 0.027 | 0.051 |
| **MCE of bias of** $\boldsymbol{S}_{\boldsymbol{boot}}$ | 0.008 | 0.014 | 0.023 |
| $\boldsymbol{0.7\leq}\boldsymbol{C}_{\boldsymbol{pop}}\boldsymbol{<0.75}$ **(4,117 scenarios)** | | | |
| **MCE of bias of** $\boldsymbol{S}_{\boldsymbol{VH}}$ | 0.007 | 0.017 | 0.025 |
| **MCE of bias of** $\boldsymbol{S}_{\boldsymbol{boot}}$ | 0.006 | 0.010 | 0.013 |
| $\boldsymbol{0.75\leq}\boldsymbol{C}_{\boldsymbol{pop}}\boldsymbol{<0.8}$ **(4,323 scenarios)** | | | |
| **MCE of bias of** $\boldsymbol{S}_{\boldsymbol{VH}}$ | 0.006 | 0.013 | 0.018 |
| **MCE of bias of** $\boldsymbol{S}_{\boldsymbol{boot}}$ | 0.005 | 0.007 | 0.009 |
| $\boldsymbol{0.8\leq}\boldsymbol{C}_{\boldsymbol{pop}}\boldsymbol{<0.85}$ **(884 scenarios)** | | | |
| **MCE of bias of** $\boldsymbol{S}_{\boldsymbol{VH}}$ | 0.005 | 0.013 | 0.016 |
| **MCE of bias of** $\boldsymbol{S}_{\boldsymbol{boot}}$ | 0.004 | 0.006 | 0.007 |
| $\boldsymbol{0.85\leq}\boldsymbol{C}_{\boldsymbol{pop}}\boldsymbol{<0.9}$ **(4 scenarios)** | | | |
| **MCE of bias of** $\boldsymbol{S}_{\boldsymbol{VH}}$ | 0.007 | 0.011 | 0.013 |
| **MCE of bias of** $\boldsymbol{S}_{\boldsymbol{boot}}$ | 0.005 | 0.005 | 0.005 |

*Supplementary Table 5 - Simulation Study 2: The 2.5^th^, 25^th^, 50^th^, 75^th^ and 97.5^th^ percentiles of* $mean\left( S_{pop} \right)$ *across scenarios where data was simulated with a zero covariance structure, stratified by* $C_{pop}$

|  | **2.5%** | **25%** | **50%** | **75%** | **97.5%** | **Mean bias (sd)** | **Mean magnitude of bias (sd)** |
| --- | --- | --- | --- | --- | --- | --- | --- |
| **Scenarios where** $\boldsymbol{N}_{\boldsymbol{adapted}}$ **converged (11,772 scenarios)** | | | | | | | |
| $\boldsymbol{N}_{\boldsymbol{original}}$ | 0.845 | 0.868 | 0.884 | 0.905 | 0.993 | -0.008 (0.045) | 0.030 (0.035) |
| $\boldsymbol{N}_{\boldsymbol{adapted}}$ | 0.881 | 0.896 | 0.900 | 0.905 | 0.922 | 0.000 (0.048) | 0.004 (0.047) |
| $\boldsymbol{0.6\leq}\boldsymbol{C}_{\boldsymbol{pop}}\boldsymbol{<0.65}$ **(1,303 scenarios)** | | | | | | | |
| $\boldsymbol{N}_{\boldsymbol{original}}$ | 0.899 | 0.916 | 0.928 | 0.947 | 1.011 | 0.037 (0.046) | 0.038 (0.045) |
| $\boldsymbol{N}_{\boldsymbol{adapted}}$ | 0.861 | 0.893 | 0.902 | 0.909 | 0.959 | 0.002 (0.053) | 0.008 (0.050) |
| $\boldsymbol{0.65\leq}\boldsymbol{C}_{\boldsymbol{pop}}\boldsymbol{<0.7}$ **(2,138 scenarios)** | | | | | | | |
| $\boldsymbol{N}_{\boldsymbol{original}}$ | 0.885 | 0.896 | 0.904 | 0.913 | 0.939 | 0.006 (0.014) | 0.011 (0.011) |
| $\boldsymbol{N}_{\boldsymbol{adapted}}$ | 0.883 | 0.895 | 0.900 | 0.906 | 0.918 | 0.000 (0.009) | 0.005 (0.006) |
| $\boldsymbol{0.7\leq}\boldsymbol{C}_{\boldsymbol{pop}}\boldsymbol{<0.75}$ **(3,403 scenarios)** | | | | | | | |
| $\boldsymbol{N}_{\boldsymbol{original}}$ | 0.868 | 0.878 | 0.884 | 0.890 | 0.907 | -0.015 (0.010) | 0.016 (0.008) |
| $\boldsymbol{N}_{\boldsymbol{adapted}}$ | 0.888 | 0.896 | 0.900 | 0.904 | 0.912 | 0.000 (0.006) | 0.004 (0.004) |
| $\boldsymbol{0.75\leq}\boldsymbol{C}_{\boldsymbol{pop}}\boldsymbol{<0.8}$ **(3,538 scenarios)** | | | | | | | |
| $\boldsymbol{N}_{\boldsymbol{original}}$ | 0.850 | 0.860 | 0.866 | 0.872 | 0.886 | -0.033 (0.009) | 0.033 (0.009) |
| $\boldsymbol{N}_{\boldsymbol{adapted}}$ | 0.890 | 0.897 | 0.900 | 0.904 | 0.910 | 0.000 (0.005) | 0.003 (0.003) |
| $\boldsymbol{0.8\leq}\boldsymbol{C}_{\boldsymbol{pop}}\boldsymbol{<0.85}$ **(781 scenarios)** | | | | | | | |
| $\boldsymbol{N}_{\boldsymbol{original}}$ | 0.829 | 0.842 | 0.848 | 0.854 | 0.867 | -0.052 (0.010) | 0.052 (0.010) |
| $\boldsymbol{N}_{\boldsymbol{adapted}}$ | 0.890 | 0.897 | 0.900 | 0.903 | 0.909 | -0.000 (0.005) | 0.003 (0.003) |
| $\boldsymbol{0.85\leq}\boldsymbol{C}_{\boldsymbol{pop}}\boldsymbol{<0.9}$ **(3 scenarios)** | | | | | | | |
| $\boldsymbol{N}_{\boldsymbol{original}}$ | 0.802 | 0.806 | 0.809 | 0.819 | 0.829 | -0.086 (0.014) | 0.086 (0.014) |
| $\boldsymbol{N}_{\boldsymbol{adapted}}$ | 0.890 | 0.894 | 0.899 | 0.900 | 0.901 | -0.001 (0.006) | 0.001 (0.005) |
| **Scenarios where** $\boldsymbol{N}_{\boldsymbol{adapted}}$ **did not converge (195 scenarios)** | | | | | | | |
| $\boldsymbol{N}_{\boldsymbol{original}}$ | 0.872 | 1.027 | 1.138 | 1.189 | 1.353 | 0.217 (0.162) | 0.235 (0.134) |
| $\boldsymbol{N}_{\boldsymbol{adapted}}$ | NA | NA | NA | NA | NA | NA | NA |
